# Metabolic Engineering and Bioprocess Optimization Strategies Promote l‑Azidohomoalanine Biosynthesis in Fed-Batch Bioreactor Culture

**DOI:** 10.1021/acsomega.6c05952

**Published:** 2026-09-29

**Authors:** Claudia Lacombe, Gerald Striedner, Birgit Wiltschi

**Affiliations:** † Department of Biotechnology and Food Sciences, Institute for Bioprocess Science and Engineering, 27270 BOKU University, Vienna 1190, Austria; ‡ 298883 acib—Austrian Centre of Industrial Biotechnology, Vienna 1190, Austria

## Abstract

The noncanonical amino acid l-azidohomoalanine (Aha) can replace methionine in ribosomal translation under tightly controlled conditions. Aha has been commonly employed to functionalize proteins by the bioorthogonal conjugation with alkynes, the so-called “click chemistry”. However, its application for scalable recombinant protein production is currently hampered by the high price. To address this shortcoming, a biosynthesis cascade from inexpensive sodium azide (NaN_3_) as the precursor had been reported in the literature. Here, we advance the approach to a scalable bioprocess for the biosynthesis of Aha in *Escherichia coli*. We biosynthesized Aha via a two-enzyme cascade reaction using the cellular metabolite l-homoserine and NaN_3_ as the precursors. First, l-homoserine-*O*-acetyltransferase acetylates l-homoserine, then *O*-acetyl-l-homoserine sulfhydrylase catalyzes the conversion of the activated *O*-acetyl-l-homoserine to Aha by the nucleophilic attack of NaN_3_. In contrast to earlier studies, we focused on optimizing the biosynthesis of Aha in fed-batch bioreactor cultures of *E. coli* independent of its incorporation into target proteins. We established methods for the quantitative analysis of NaN_3_, the *O*-acetyl-l-homoserine intermediate and Aha to monitor the biosynthesis reaction. Combining gene-, strain- and process-engineering, we achieved a maximum Aha production of 1.4 mM or 0.05 mmol per gram of cell dry mass. Our study advances the biosynthesis of Aha to the next level as it demonstrates that the concentration routinely used for the global replacement of methionine can be exceeded by approximately three times. Nevertheless, further optimization is required to improve the conversion of the toxic precursor NaN_3_ such that the cobiosynthesis of Aha and its direct incorporation into a protein of interest become viable for scalable applications.

## Graphical abstract

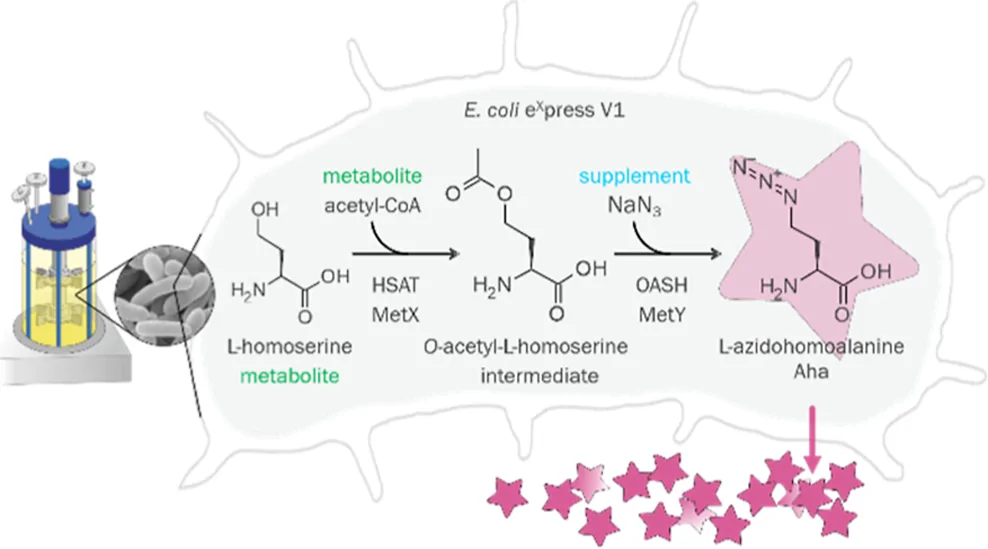

## Introduction

The standard genetic code combines four nucleotides in 64 codons to govern the biosynthesis of proteins from a canonical set of 20 amino acids. The limited side-chain diversity of the canonical amino acids has prompted the expansion of this central cipher of life beyond its evolutionary boundaries. Numerous noncanonical amino acids (ncAAs) have been incorporated into recombinant proteins, which has broadened the landscape of protein engineering.^1–6^ Hundreds of ncAAs are now available to introduce chemical, structural and functional diversity into recombinant proteins.^5,7^ Although ncAAs have become essential tools for chemistry and biology,^2,8–10^ their scalable application is often hampered by their availability and cost.^11,12^ Most ncAAs are chemically synthesized. However, the chemical synthesis of ncAAs is not sustainable and challenged by uncertain costs due to the shortage of raw materials.^13^ Biosynthetic pathways for ncAAs appear an innovative solution to address these challenges. Despite the fact that more than 700 ncAAs occur naturally,^14^ their biosynthesis pathways are largely unknown. Nevertheless, biosynthesis pathways for a small number of biotechnologically relevant ncAAs were previously reported.^15^ Provided that it is efficient, the biosynthesis approach bears the potential to facilitate the sustainable production of ncAAs at sufficient quantities.^13^

In this study, we embarked on the biosynthesis of an ncAA that carries a unique biorthogonal chemical group and can be used directly in protein translation under controlled conditions. l-azidohomoalanine (Aha) combines these outstanding properties: it carries a bioorthogonal azide moiety in its side chain, can be biosynthesized and has been incorporated routinely into recombinant proteins in place of methionine (Met,^16^ reviewed by Marin et al.^17^). In fact, Aha has become one of the most important bioorthogonal tags for a wide variety of applications in biotechnology such as labeling, imaging or engineered proteins and materials.^18–23^ Aha’s azide moiety reacts spontaneously with ring-constrained cyclooctynes, such as the dibenzocyclooctyne group^24^ while the reaction with terminal unconstrained alkynes occurs only in the presence of copper­(I) as the catalyst.^25^ The strain-promoted azido-alkyne cycloaddition reactions are considered bioorthogonal because they occur without interfering with naturally occurring biomolecules.^24,26,27^ Aha has been exploited to study newly synthesized proteins^28,29^ and Aha-labeled proteins can be selectively bound to antibodies or other affinity molecules, peptides, biomaterials or polymers.^21,30–32^ Being an analog of Met, Aha has been incorporated predominantly in Met auxotrophic hosts by the so-called supplementation-based incorporation method^16,33^ (SPI) (reviewed by Marin et al. (2025)^17^). This approach globally reassigns the Met ATG codon to Aha and the selectivity of its incorporation depends on the occurrence and position of the ATG codon in the coding sequence. Indeed, Aha is readily tolerated as a Met surrogate by the methionyl-tRNA synthetases of bacteria, yeast and mammalian cells.^16,34,35^ Recently, Koch et al. devised a pyrrolysyl-tRNA synthetase mutant that can incorporate Aha site-specifically at an in-frame amber stop codon. However, the mutant enzyme promiscuously accepts Met as a substrate albeit to a lower extent than Aha.^36^

Budisa and co-workers described a two-step biosynthesis pathway for Aha in *Escherichia coli*.^37^ The first enzyme, l-homoserine-*O*-acetyltransferase (MetX) uses the cellular metabolites acetyl-CoA and l-homoserine (Hse) to produce *O*-acetyl-l-homoserine (OAH) as a reactive intermediate. The second enzyme *O*-acetyl-l-homoserine sulfhydrylase (MetY) converts OAH and sodium azide (NaN_3_) to Aha (Figure 1). The simultaneous biosynthesis and incorporation of Aha into target proteins in *E. coli* have been previously demonstrated.^37,38^ However, the titers of Aha-labeled protein were below 50 mg/L and the concentration of the biosynthesized Aha has not been assessed yet.

**Figure 1:**
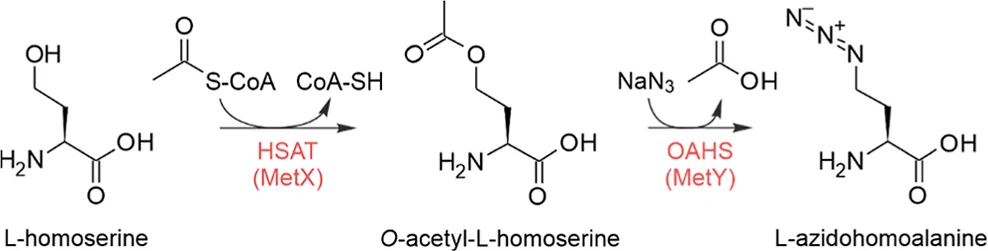
.Schematic illustration of the l-azidohomoalanine biosynthesis cascade.

We believe that monitoring the Aha biosynthesis independently of its incorporation into proteins could provide valuable insights for a subsequent improvement of the protein labeling efficiency with biosynthesized Aha. In this study, we combined gene-, strain- and process-engineering to systematically improve Aha biosynthesis in *E. coli*. Employing varied gene syntaxes, we modulated the expression of *metX* and *metY* such that the flux through the Aha biosynthesis pathway was optimized. The *E. coli* e^X^press strain had been reported previously to improve recombinant protein production by decoupling it from growth.^39,40^ This strain proved advantageous for the high-level production of the cascade enzymes MetX and MetY in comparison to the reference *E. coli* strain BL21­(DE3). A MetX variant that is insensitive against OAH feedback inhibition^41^ further elevated Aha productivity. To our best knowledge, the current study first reports the upscaling of Aha’s biotechnological production from shake flask to fed-batch bioreactor cultures. We monitored the cascade reaction using different analytics, in particular the qualitative and quantitative analysis of NaN_3_, the intermediate product OAH and Aha. Guided by the analytics, we fine-tuned the feeding regime and the production time to maximally convert NaN_3_ to Aha. We found that the optimized production strain biosynthesized Aha at a maximal titer of 1.4 mmol/L under optimal bioprocess conditions.

## Materials and Methods

All compounds were purchased at Carl Roth (Karlsruhe, Germany), Sigma-Aldrich (St. Louis, MO) or Merck KGaA (Darmstadt, Germany) unless indicated otherwise. Gibco Bacto yeast extract (LOT 2461100) was purchased from ThermoFisher Scientific (Waltham, MA) and the noncanonical amino acids were from Iris Biotech (Marktredwitz, Germany).

### Strains and Plasmids

Aha was biosynthesized in three different *E. coli* strains. EnGenes e^X^press strains V1 (BL21­(DE3)::TN7⟨pAraB-*gp2*⟩Δ*araBADC*) and V2 (BL21-AI⟨*gp2*⟩) were compared to BL21­(DE3) (*E. coli* B F^–^ *ompT hsdS*_B_(r_B_^–^ m_B_^–^)*dcm* ^+^ *gal* λ­(DE3);^42^ Merck KGaA, Darmstadt, Germany) as the benchmark expression strain. The generation of the growth-decoupled strains e^X^press V1 and V2 was previously described by Stargardt et al.^39,40^ *E. coli* strain NEB5α (New England Biolabs GmbH (NEB), Ipswitch, MA) was used for the cloning steps. Q5 high-fidelity DNA polymerase (NEB) was employed for PCR amplification of DNA fragments and all restriction enzymes for the cloning procedures were from NEB. The plasmids were constructed using type II S restriction cloning. Briefly, the constructs were assembled from five DNA fragments carrying compatible 4-base overhangs for directed assembly using BsaI-HFv2 (NEB) and T4 DNA ligase (NEB) in a one-pot reaction. The amino acid sequences of MetX from *Deinococcus radiodurans* (genbank accession Q9RVZ8) and MetY from *Bacillus stearothermophilus* (genbank accession E16859.1) were reverse-translated using the codon scrambler software^43^ and ordered as clonal genes (Twist Bioscience, CA, USA). All fragments for the assembly reaction were PCR-amplified using the primers listed in Table S1 and gel purified (Monarch DNA Gel Extraction Kit, NEB). Plasmids pXYI, pXYII, pXYIII were assembled using fragments with compatible overhangs (Figure 2). To generate pX*YIII, we excised the *metX* gene from pXYIII using NdeI and AatII and replaced it with the mutant allele *metX L218A* (Table S2) cut with the same enzymes. The insert *metX L218A* was purchased in gBlock format from Integrated DNA Technologies (IDT, Coralville, IA). BL21­(DE3) as well as the e^X^press strain V1 and V2 were transformed with the plasmids by electroporation using a BTX ECM 630 electroporator (BTX, Holliston, MA) set at 1800 V, 200 Ω, 25 μF in a 0.1 cm electroporation cuvette (Biozym, Vienna, Austria). All plasmid sequences were confirmed by Sanger sequencing (Microsynth, Vienna, Austria). Master and working cell banks were prepared using M9ZB complex medium and semisynthetic medium with d-glucose containing 50 mg/L kanamycin each for plasmid maintenance. The ingredients of the M9ZB and the semisynthetic medium were previously described.^44,45^

**Figure 2:**
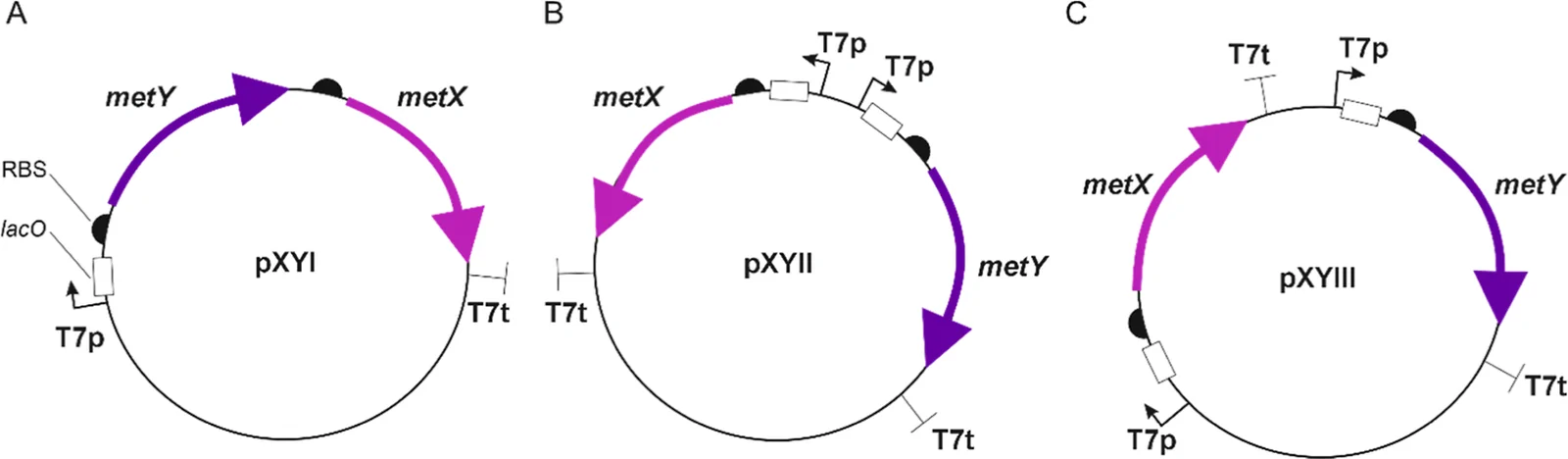
Plasmid architectures for the expression of the Aha biosynthesis genes *metXY*. The recombinant plasmid carries *metXY*, which encodes MetX and MetY. (A) Plasmid pXYI carrying a bicistronic expression cassette of *metXY* under the control of a single IPTG-inducible T7_*lacO*_ promoter (T7p) and T7 terminator (T7t). The translation of each open reading frame was controlled by an individual ribosome binding site (RBS); (B) plasmid pXYII carried divergent monocistronic expression cassettes of *metX* and *metY*, each under control of T7p and T7t; (C) plasmid pXYIII with two monocistronic T7p*-metX*-T7t and T7p*-metY*-T7t expression cassettes in tandem.

### Biosynthesis of Aha in Fed-Batch-like μ-Bioreactor Cultures

The cultures were performed in the BioLector microfermentation system (fed-batch-like μ-bioreactor cultures) in 48-well Flowerplates (m2p-laboratories GmbH, Baesweiler, Germany) as described by Stargardt et al.^40^ employing the following changes: initially, the medium in each well was inoculated with cells to an initial attenuance at 600 nm (*D*_600 start_) of 0.1 as inoculation. The batch phase was carried out using 1% (w/v) d-glucose. The fed-batch phase was operated with enzymatic release of d-glucose from dextran using 0.6% (v/v) of enzyme mix (EnzMix, m2p-laboratories GmbH). 1 mM isopropyl β-d-1-thiogalactopyranoside (IPTG) (GERBU Biotechnik, Heidelberg, Germany) was used for the induction of gene expression in all strains. 10 mM l-arabinose (Sigma-Aldrich, Saint Louis, MO) was used to induce the expression of *gp2* in the e^X^press strains.^39,40^ Cells were grown in batch mode for 15 h. At induction of *metXY*, NaN_3_ was added in pulses every hour to reach a total addition of 5 μmol. For the analysis of different feedstocks in μ-bioreactor cultures, the compounds were added at the induction of *metXY* as a pulse feed at the concentration indicated in the result section. Biomass was continuously monitored every 15 min using scattered light detection at 620 nm, and the cell dry mass (CDM) was derived from a predefined calibration curve. 1 mg of CDM was collected for the analysis by sodium dodecyl sulfate polyacrylamide gel electrophoresis (SDS-PAGE, PA-gels were from ThermoFisher Scientific). SDS-PA gels were stained using Coomassie blue stain as previously described.^39^

### Biosynthesis of Aha in Fed-Batch Bioreactor Cultures

The fed-batch fermentation cultures were cultivated in computer-controlled Dasgip Parallel Bioreactor Systems (Eppendorf SE, Hamburg, Germany). The media compositions are indicated in Tables S4 and S5. Kanamycin was not added during the production phases in μ-bioreactor (see above) and fed-batch bioreactor cultures. The cells were precultured in semisynthetic medium with d-glucose containing 50 mg/L kanamycin in 250 mL baffled shake-flasks. The cultures were inoculated with the working cell bank to a *D*_600 start_ of 0.1 and incubated at 37 °C in an orbital shaker at 200 rpm until they reached a *D*_600_ of 3. 400 mL of batch medium (composition Table S4) were supplemented with 2 mL of 1:10 diluted antifoam (Struktol J673A, Schill Seilacher, Germany) prior to inoculation. Foaming was further controlled by the addition of the antifoam based on a short circuit-driven level sensor. The batch culture medium was inoculated with the preculture to an initial *D*_600_ of 0.08. The batch phase was conducted at 37 ± 0.2 °C until the CDM reached 10 g/L in 400 mL. The pH was set to pH 7 ± 0.2 by addition of 12.5% (v/v) NH_3_. The fed-batch phase at 30 ± 0.2 °C was separated into two phases: A first exponential feed phase at a growth rate of 0.1 h^–1^ for 21 h was followed by a linear feed phase at a growth rate ranging from 0.04 to 0.03 h^–1^ for 12 h. The calculated CDM at the end of fermentation was 45 g/L unless stated otherwise. Gene expression was induced at the beginning of the linear feed using 1 mM IPTG (*metXY*) and 10 mM l-arabinose (*gp2*) only for the e^X^press strains. At induction, the 360 mM NaN_3_ stock solution was added to the culture as a linear feed to reach 20 to 50 mmol NaN_3_ at the end of the fermentation. pH and dissolved oxygen (DO) were continuously measured with probes from Hamilton (Bonaduz AG, Switzerland). DO was maintained above 30% saturation by adjusting the stirrer speed and the air flow.

Cell growth was monitored off-line by measuring the *D*_600_ in a DiluPhotometer (Implen, Munich, Germany). To assess the CDM, 2 mL polypropylene tubes (Eppendorf) were dried at 105 °C for 48 h, cooled to room temperature for 4 h in a desiccator and weighed. 1 mL culture was sampled and centrifuged in the previously weighed 1.5 mL tubes using a tabletop centrifuge (Eppendorf; 10 min, 4 °C, 16,000*g*). The cell pellets were washed twice with 1.8 and 1.6 mL deionized H_2_O, centrifuged as described and the supernatants were discarded. The pellets were incubated at 105 °C for 48 h, cooled for 4 h in a desiccator and weighed. Soluble and insoluble protein fractions were analyzed according to Fink et al.^46^ by SDS-PAGE on 4–12% Bis-Tris gels (ThermoFisher Scientific) if not indicated otherwise. A mix of 65% (v/v) protein sample, 25% (v/v) 4× NuPAGE LDS sample buffer (ThermoFisher Scientific) and 10% (v/v) 10× NuPAGE reducing agent (ThermoFisher Scientific) was heated at 95 °C for 10 min prior to loading onto the gel. The gel was stained using Coomassie R250. The product concentration was estimated by densitometry using ImageJ-Fiji (version v1-54i)^47^ and bovine serum albumin (Sigma-Aldrich) as the quantification standard.

### Analytical Methods to Assess Aha, OAH and NaN_3_ Levels

Aha production was qualitatively analyzed by thin-layer chromatography using normal phase HPTLC-plates ALUGRAM Xtra Nano-SILGUR/UV254 with nano silica gel and concentration zone (Carl Roth). The TLC was developed in a mixture of chloroform, 96% (v/v) EtOH, acetic acid and 25% (v/v) NH_3_ at a ratio of 5:2.6:0.9:0.6 (v/v) in a rectangular TLC developing tank (Sigma-Aldrich). The plate was incubated in ethanolic ninhydrin solution (3% w/v) for 30 s. Compound spots were visualized after incubation of the plate for 10 min at 105 °C.

To assess the residual azide concentration, 20 μL of 0 to 10 mM NaN_3_ in water (for calibration) or medium supernatant were added to 180 μL of 50 mM FeCl_3_ in 10 mM HCl in a 96-well plate. After 5 min of incubation at room temperature, the absorbance of the ferric azide complex was determined at 450 nm using a spectrophotometer. The calibration curve was done in parallel to the samples of unknown concentration and each measurement was performed in triplicate.

In the μ-bioreactor cultures, Aha and OAH were quantified using precolumn derivatization with *o*-phthalaldehyde followed by high performance liquid chromatography separation (OPA-HPLC) on an InfinityLab Poroshell HPH-C18 (693975-502, 3.0 × 150 mm, 2.7 μm, 100 Å pore size) solvent saver column (Agilent, Santa Clara, CA). The samples were spiked with a final concentration of 2.5 mg/L 3-(2-thienyl-dl-alanine) as an internal standard for the derivatization. Prior to injection, the samples were filtered using a 0.2 μm pore size Whatman Puradisc syringe filter (Sigma-Aldrich). The protocol for the separation of amino acids by OPA-HPLC was as described by Széliová et al.^48^ Solvent A (10 mM of K_2_HPO_4_, 10 mM of K_2_B_4_O_7_·4H_2_O, pH 8.2 ± 0.05) and solvent B (45% (v/v) CH_3_OH, 45% (v/v) CH_3_CN, 10% (v/v) H_2_O) were prepared with ultrapure water from a Milli-Q system (Merck Millipore). Buffers were filtered (0.2 μm pore size, Sigma-Aldrich) and degassed prior to use. Samples were analyzed using a gradient elution from 2 to 57% solvent B over 23 min, followed by a step to 100% solvent B and re-equilibration at 2% solvent B. The total run time was 45 min. OPA fluorescence was monitored at 345 and 455 nm for excitation and emission wavelengths, respectively. The calibration standards 0, 25, 50, 75, 100, 125, and 150 mg/L Aha were prepared in μ-bioreactor culture medium diluted 1:4 with deionized H_2_O. The culture medium had been collected at the end of the μ-bioreactor culture with NaN_3_ but without induction of *metXY*. Likewise, the calibration standards 0, 60, 120, 180, 240, 300, and 360 mg/L OAH were prepared.

In fed-batch bioreactor cultures, Aha was determined by mass spectrometry (MS). The samples were diluted 1:2 with 10% (v/v) acetic acid, centrifuged and the supernatant was collected for further analysis. The calibration curve was prepared with 0, 8, 40, 200, and 100 μM Aha in water using an internal standard (isotope labeled glutamine). 1 μL was injected of each sample for analysis by MS.

## Results and Discussion

### Gene Syntax Design to Enhance the Flux through the Biosynthesis Cascade toward Aha

Previously, the biosynthesis of Aha was published along its direct incorporation into recombinant target proteins by Schipp et al.^37^ They transformed the Met auxotrophic MDS15A *E. coli* strain with two different vectors. The first vector carried the biosynthesis genes, *metX* and *metY* both from *Corynebacterium glutamicum*. The second vector carried the gene encoding the recombinant target protein. Aha was biosynthesized and incorporated into the target protein in the same culture. Although appealing, this strategy complicates the control of the bioprocess. The incorporation of Aha occurs residue-specifically in a Met auxotrophic *E. coli* strain. In shake flask cultures, the cells are grown to late log phase in medium containing a limiting concentration of Met and the target gene expression is induced as soon as Met is depleted. Usually, 0.5–1 mM synthetic Aha are added at induction (reviewed by Marin et al.^17^). For an efficient SPI of Aha, its biosynthesis must be synchronized with the depletion of Met such that the intracellular Aha concentration solidly exceeds that of Met at the induction of the target gene expression. Efficient Aha biosynthesis requires a steady supply of the metabolic precursors Hse and OAH, which calls for balanced and optimized routing of glucose-derived flux into the Hse and acetyl-CoA pathways. Metabolic engineering offers a route to achieve this, as evidenced by improvements in Hse and OAH titers in *E. coli*.^41,49^ To dissect these aspects in our study, we focused on the biosynthesis of Aha and did not directly incorporate it into a target protein. This strategy allowed us to control the process parameters and establish analytics to monitor the Aha biosynthesis. In particular, we aimed at modulating gene expression and protein production to achieve a balanced stoichiometry of the cascade reaction. We hypothesized that the flux through the cascade would be maximal if the two enzyme activities and the supply with the precursors were balanced. We chose the *metX* sequence from *D. radiodurans* and the *metY* sequence from *B. stearothermophilus* (Table S2). We codon harmonized^43^ the sequences for expression in *E. coli* to preserve the translation rhythm from the source organisms. Since the gene syntax affects expression,^50^ we generated three expression constructs that differed in the mono- or bi-cistronic expression of *metX* and *metY* along with their mutual orientation (Figure 2). To keep the metabolic burden of the cells as low as possible, we encoded both genes on a single plasmid that contained a basis of mobility element to improve its stability.^51^

### The Monocistronic Tandem Expression of *metX* and *metY* in a Growth-Decoupled *E. coli* Protein Production Strain Enhanced Aha Biosynthesis

We transformed three different *E. coli* strains with the *metXY* expression plasmids, which resulted in nine different strain/plasmid combinations (Table S6). BL21­(DE3) is a commonly used *E. coli* strain for recombinant protein production^52^ and we designated it as the reference strain in this study. The *E. coli* e^X^press strains V1 and V2 were used to optimize recombinant protein production in a nongrowing host.^39,40^ The e^X^press strains V1 and V2 derive from the *E. coli* strains BL21­(DE3) and BL21-AI. A main difference between them concerns the regulation of the expression of the T7 RNA polymerase (T7 RNAP) gene. In e^X^press V1, the T7 RNAP gene is under control of the *lac*UV5 promoter. In e^X^press V2, its expression is controlled by the l-arabinose inducible *araBAD* promoter. *gp2* is under the control of the arabinose-inducible *araBAD* promoter in both strains. Both strains exploit T7 RNAP and the Gp2 protein from bacteriophage T7 to constitute an orthogonal transcription system. T7 RNAP initiates transcription only from a T7 promoter but not from *E. coli* host promoters.^42^ Gp2 selectively inhibits *E. coli* RNA polymerase but not T7 RNAP.^53–55^ If coexpressed, Gp2 prevents the *de novo* transcription of host genes while T7 RNAP transcribes the gene(s) of interest at high level. Since the host cells cease to grow without *de novo* protein synthesis, the cellular resources freed by the growth retardation can be channeled effectively into the accumulation of the recombinant protein(s) of interest.^39,40^ In previous studies, this led to increased transcription of the gene(s) of interest such that the recombinant protein(s) amounted to 55% of cell dry mass (CDM). The yield of total as well as soluble protein fractions was elevated more than 3-fold in comparison to *E. coli* BL21­(DE3).^39^ We hypothesized that decoupling growth from MetXY production would reduce the metabolic burden, increase the level of functional MetXY and thus enhance Aha biosynthesis. NaN_3_ inhibits *E. coli* cell growth by disrupting protein export and impairing respiratory processes.^56–59^ Decoupling growth and the biosynthesis of Aha from NaN_3_ as the precursor would presumably minimize the toxic effects of NaN_3_ because we could add it after the growth phase.

We compared the efficiency of Aha biosynthesis between the nine strain/plasmid combinations in automated fed-batch-like μ-bioreactor cultures. The cells were cultivated in enzymatic glucose release medium to simulate a fed-batch-like process without external substrate feed. NaN_3_ was added in pulses every hour resulting in a cumulative addition of 5 μmol, which corresponded to a final concentration of up to 6 mM. This dosage was in agreement with the NaN_3_ supplementation reported in the literature,^59^ which had been optimized to balance Aha production while maintaining cell viability.^37^ We analyzed the production of MetX and MetY (Figure S1) and the biosynthesis of Aha at 8 h postinduction of *metXY* (Figures 3 and S4). Out of the nine strain/plasmid combinations, BL21­(DE3)­{pXYIII}, e^X^press V1­{pXYII}, e^X^press V1­{pXYIII} and e^X^press V2­{pXYIII} produced MetXY as indicated by the SDS-PAGE analysis (Figure S1). Notably, *metY* was more pronouncedly expressed than *metX* both from the mono- and bicistronic gene expression syntaxes (Figure S1). Independent of the *E. coli* strain, the monocistronic tandem architecture (pXYIII) yielded the highest *metXY* levels (Figure S1). We used thin-layer chromatography (TLC) as a straightforward method to qualitatively analyze the Aha production in multiple samples in parallel.^38^ According to the analytical TLC (Figure S4), *E. coli* e^X^press V1{pXYIII} appeared to most efficiently biosynthesize Aha. This finding was consistent with the *metX* expression levels, which were highest in e^X^press V1­{pXYIII} compared to the other strains (Figure S1). Next, we established a reversed-phase HPLC method (the experimental details are described in the Materials and Methods section) to quantify Aha and OAH in the culture supernatant of shake-flask cultures and fed-batch-like μ-bioreactor cultures. The HPLC results confirmed the findings of the TLC analysis. The most productive strain reached 1.2 mM Aha, whereas the other strains produced substantially lower concentrations (0.2 and 0.1 mM). When normalized to biomass, strain e^X^press V1­{pXYIII} showed a markedly higher specific productivity (0.54 mmol/g_CDM_) compared to e^X^press V2­{pXYIII} (0.06) and BL21­(DE3)­{pXYIII} (0.03) (Figure 3), indicating that the improved volumetric production is primarily driven by enhanced cellular efficiency rather than biomass accumulation. This pattern mirrored the NaN_3_ supplementation (mmol/g_CDM_), which was lowest for strain BL21­(DE3)­{pXYIII} and highest for e^X^press V1­{pXYIII} (Figure 3). As expected, cell growth was markedly inhibited after induction of *gp2* expression in the e^X^press strains compared to the benchmark expression strain. For this reason, the final biomass concentrations at the end of fermentation ranged from 2.3 to 3.3 and 4.1 g/L for e^X^press V1­{pXYIII}, e^X^press V2­{pXYIII} and BL21­(DE3)­{pXYIII}, respectively. However, while the NaN_3_ supplementation (mmol/g_CDM_) differed by roughly 2-fold between BL21­(DE3)­{pXYIII} and e^X^press V1­{pXYIII}, the latter strain was 18-fold more productive. These results suggest that NaN_3_ supplementation alone did not account for the Aha yield differences. The likely driver was the higher MetX levels in e^X^press V1­{pXYIII} relative to the other strains (Figure S1).

**Figure 3:**
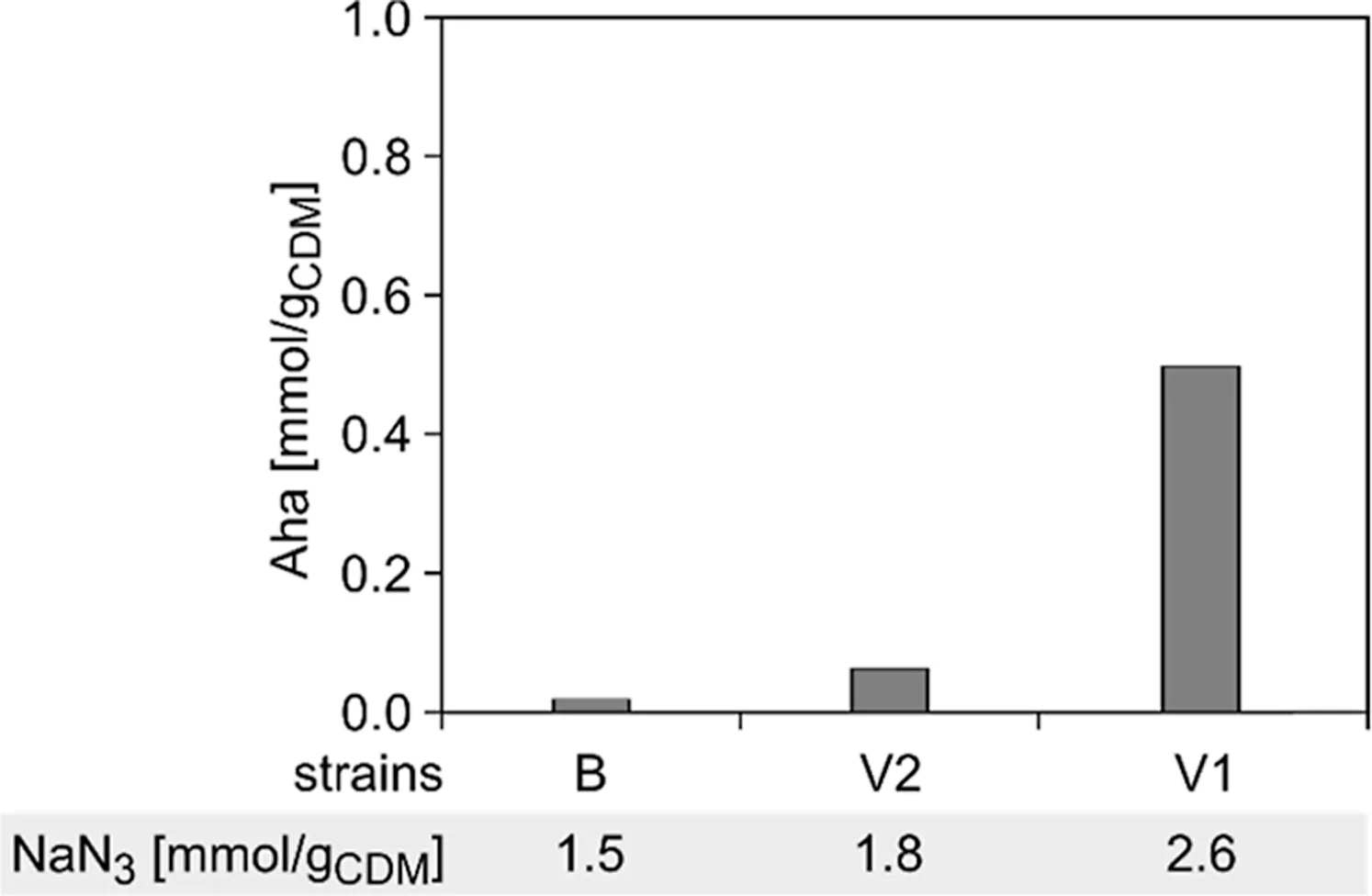
*E. coli* strain e^X^press V1­{pXYIII} behaved as the most proficient producer of Aha in fed-batch-like μ-bioreactor cultures. The Aha concentration in the culture supernatant was determined by HPLC as described in the Materials and Methods section at the end of the fermentation. Data from a representative biological experiment are shown and the results from an independent biological replicate are presented in Figure S2. All strains carried plasmid pXYIII for the expression of *metXY*. Strain designations are B, BL21­(DE3); V2, e^X^press V2; V1, e^X^press V1. At induction of *metXY*, NaN_3_ was added in pulses every hour to reach a total addition of 5 μmol. The mmol NaN_3_/g_CDM_ varied because the cultures reached different final cell densities.

### Supplementation of the Production Strain with NH_4_OAc Doubled the Biosynthesis of Aha in Fed-Batch-like μ-Bioreactor Cultures

The precursors of the cascade reaction shown in Figure 1, Hse and acetyl-CoA are produced naturally in *E. coli*. Acetyl-CoA is a central metabolite for cell growth and metabolic regulation. In the production of Aha, it serves as a substrate in the biosynthesis of Hse and acts as a cosubstrate in the MetX-catalyzed *O*-acetylation reaction. To elevate the intracellular levels of acetyl-CoA, Schipp et al. supplemented cell cultures with pantothenic acid, the precursor for the biosynthesis of acetyl-CoA.^37^ However, the addition of precursors such as pantothenic acid or Hse is rather costly beyond the shake flask scale.^60,61^ Thus, we searched for inexpensive culture supplements to further improve Aha biosynthesis.^41,49,62–65^ In *E. coli*, acetyl-CoA can be biosynthesized from d-glucose, acetate or fatty acids.^66,67^ Among these pathways, the route from acetate is most efficient, as it is short and minimizes carbon loss. Acetyl-CoA is produced from acetate either via acetyl-CoA synthetase (Acs) or through the phosphotransacetylase and acetate kinase (Pta–AckA) pathway.^66,68–70^ The Pta–AckA pathway occurs efficiently only at high extracellular acetate level. If the acetate level is low, acetyl-CoA is broken down to acetate and CoA-SH since the Pta–AckA pathway is reversible. The Acs and Pta–AckA pathways consume both 1 mol of ATP per mol of acetyl-CoA produced.^68^ Therefore, we considered that enhancing the flux through the Pta–AckA pathway by adding acetate to the medium was a promising strategy to increase acetyl-CoA availability in *E. coli*. Acetyl-CoA enters the citrate cycle where it is consumed by citrate synthase for the condensation with oxaloacetate to yield citrate. To reduce the expenditure of acetyl-CoA for citrate biosynthesis, we extended our evaluation to the supplementation of the growth medium with sodium citrate as an economically viable feedstock.^71^ We screened different acetate and citrate feeding strategies in fed-batch-like μ-bioreactor cultures. The cell cultures were pulsed with NaN_3_ every hour to reach a total addition of 5 μmol (Figure 4).

**Figure 4:**
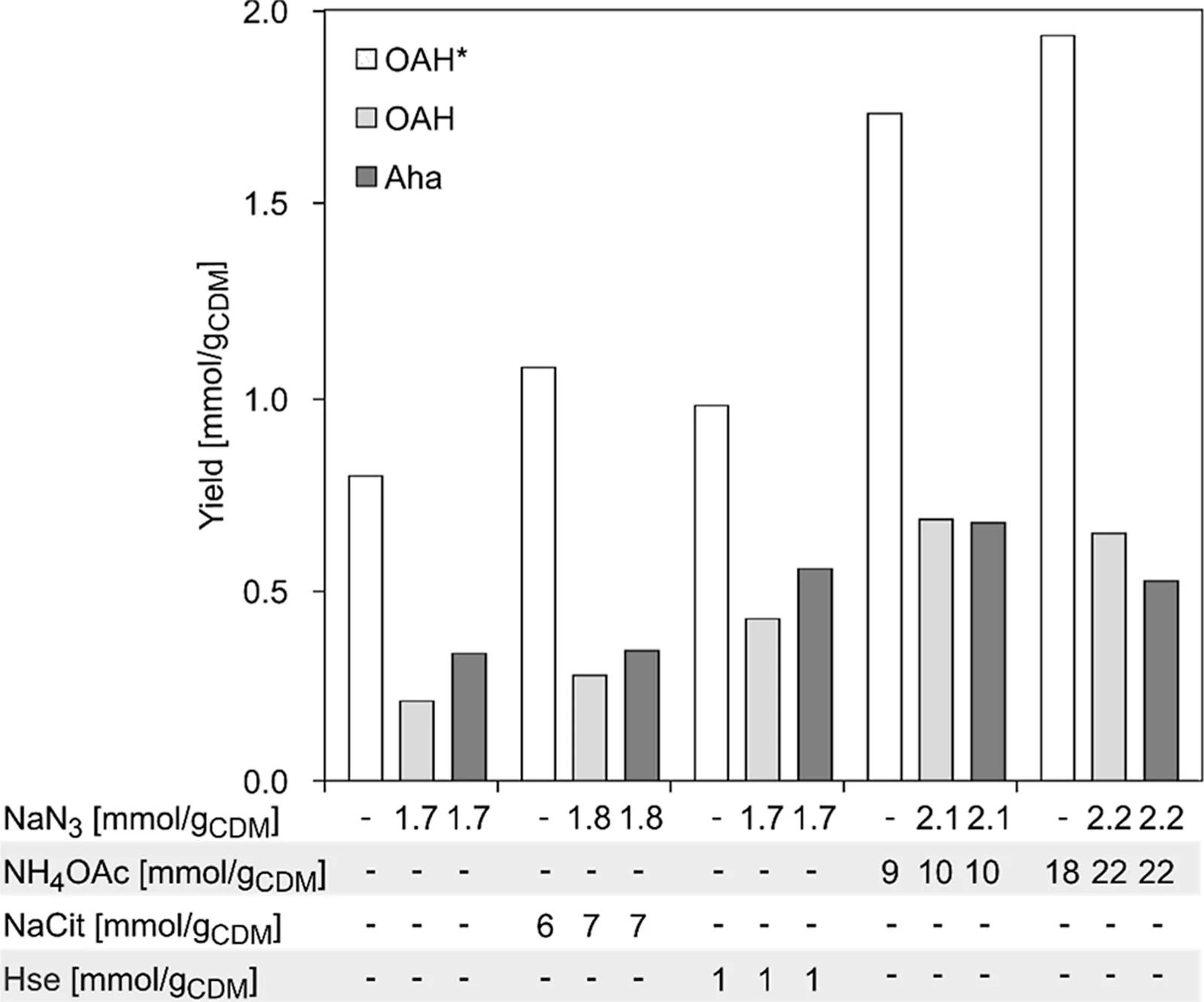
Effect of media additives during Aha production. The impact of different media additives on the production of Aha and its intermediary metabolite OAH were analyzed in fed-batch-like μ-bioreactor cultures of *E. coli* e^X^press V1 {pXYIII} 8 h postinduction of *metXY* expression. The concentration of the intermediary metabolite OAH was measured with and without (OAH*) the addition of 5 μmol NaN_3_. Refer to the Materials and Methods section for details of HPLC analysis of Aha and OAH. Data from a representative biological experiment are shown and the results from an independent biological replicate are presented in Figure S3. At induction of *metXY*, NaN_3_ was added in pulses every hour to reach a total addition of 5 μmol equaling 2 mmol/g_CDM_.

We assessed the concentration of biosynthesized Aha and its metabolic intermediate, OAH to monitor the cascade reaction. Upon induction of *metXY* expression, OAH accumulated as expected when NaN_3_ was omitted (Figure 4). We observed that the supplementation of citrate to the culture medium did not have a pronounced influence on the production of OAH nor Aha (Figure 4), and therefore we excluded it from subsequent experiments. In contrast, we found that the addition of ammonium acetate (NH_4_OAc) to the culture medium led to a pronounced increase in the Aha level. While the supplementation of the cells with 9–10 mmol NH_4_OAc/g_CDM_ led to a 2-fold increase in the production of OAH and Aha relative to the unsupplemented culture, 18–22 mmol NH_4_OAc/g_CDM_ did not further elevate it. Acetate accumulation in *E. coli* leads to intracellular acidification, which increases the metabolic burden and may inhibit cell growth.^72^ We observed a growth retardation of up to 23% in the presence of 9 to 22 mmol NH_4_OAc/g_CDM_ relative to the unsupplemented culture (Figure 4).

Next, we explored whether supplementing the cells with Hse would alleviate a potential shortage of the metabolite for Aha biosynthesis. We found that relative to the unsupplemented culture, the addition of 1 mmol Hse/g_CDM_ slightly increased the level of OAH in the absence of NaN_3_ yet doubled the production of Aha in its presence (Figure 4). This observation indicates that the reaction of OAH with NaN_3_ acts as a “sink” for OAH. If it is not consumed, it accumulates and may trigger feedback inhibition of MetX as was observed previously in *C. glutamicum*.^41^ Although it is not known whether MetX from *D. radiodurans* is also feedback inhibited by its product OAH (*vide infra*), a block in the MetX step of Aha biosynthesis presumably leads to the accumulation of Hse, the precursor of OAH (Figure 1). Kotre et al. demonstrated that Hse exerts feedback inhibition on its own biosynthesis in *E. coli* by inhibiting glutamate dehydrogenase activity.^73^ From this perspective, supplementation of Hse or elevated OAH levels, e.g. by feeding of NH_4_OAc, should be coupled to an efficient transformation of OAH into Aha for high productivity of the biosynthesis pathway. In our study, supplementation of 1 mmol Hse/g_CDM_ yielded Aha levels comparable to those obtained by feeding 9–10 mmol NH_4_OAc/g_CDM_. Since the latter is substantially less expensive than the first (approximately 1.5 orders of magnitude per gram; our own online search result^60,74^ ) NH_4_OAc represents a viable feed stock for scalable Aha biosynthesis.

To conclude, we improved the accumulation of Aha by supplying acetate as an inexpensive medium ingredient as opposed to the addition of expensive compounds such as Hse or pantothenic acid in previous reports.^37,38^

### Scaling Up from Fed-Batch-like μ-Bioreactor to 1 L Fed-Batch Bioreactor Cultures Reduced Aha Production

Next, we explored the optimal NaN_3_ feeding strategy. Since feeding fed-batch-like μ-bioreactor cultures is not practical, we scaled-up the Aha production to 1 L fed-batch bioreactor cultures and first employed continuous addition of NaN_3_. In the fed-batch-like μ-bioreactor cultures d-glucose was enzymatically released from dextran to simulate a fed-batch phase. In the 1 L fed-batch bioreactor cultures in contrast, we continuously fed d-glucose medium (Table S5) to the cells in a controlled manner to maintain a predefined growth rate (see Materials and Methods for technical details). We started the bioprocess with a batch phase for 12 h at 37 °C until the CDM reached a total of 10 g/L. Afterward, we shifted the cells to an exponential feed phase for 21 h at 30 °C with a specific growth rate of 0.1 h^–1^ targeting a final CDM of 40 g/L. Finally, the cells were subjected to a linear feeding phase for 12 h with a decreasing growth rate ranging from 0.04 to 0.03 h^–1^. The targeted CDM at the end of fermentation was 45 g/L. This final feed phase was linear because the expression of the *gp2* stalled growth as expected.^39,40^ We evaluated the three strains that had shown the highest Aha production levels in the fed-batch-like μ-bioreactor cultures and confirmed that e^X^press V1­{pXYIII} was the most suitable variant for the production of Aha (Figure 5). The lower NaN_3_-to-biomass ratio for BL21­(DE3)­{pXYIII} reflected its continued biomass accumulation during the production phase, in contrast to the growth-decoupled e^X^press V1­{pXYIII} and V2­{pXYIII} strains.

**Figure 5:**
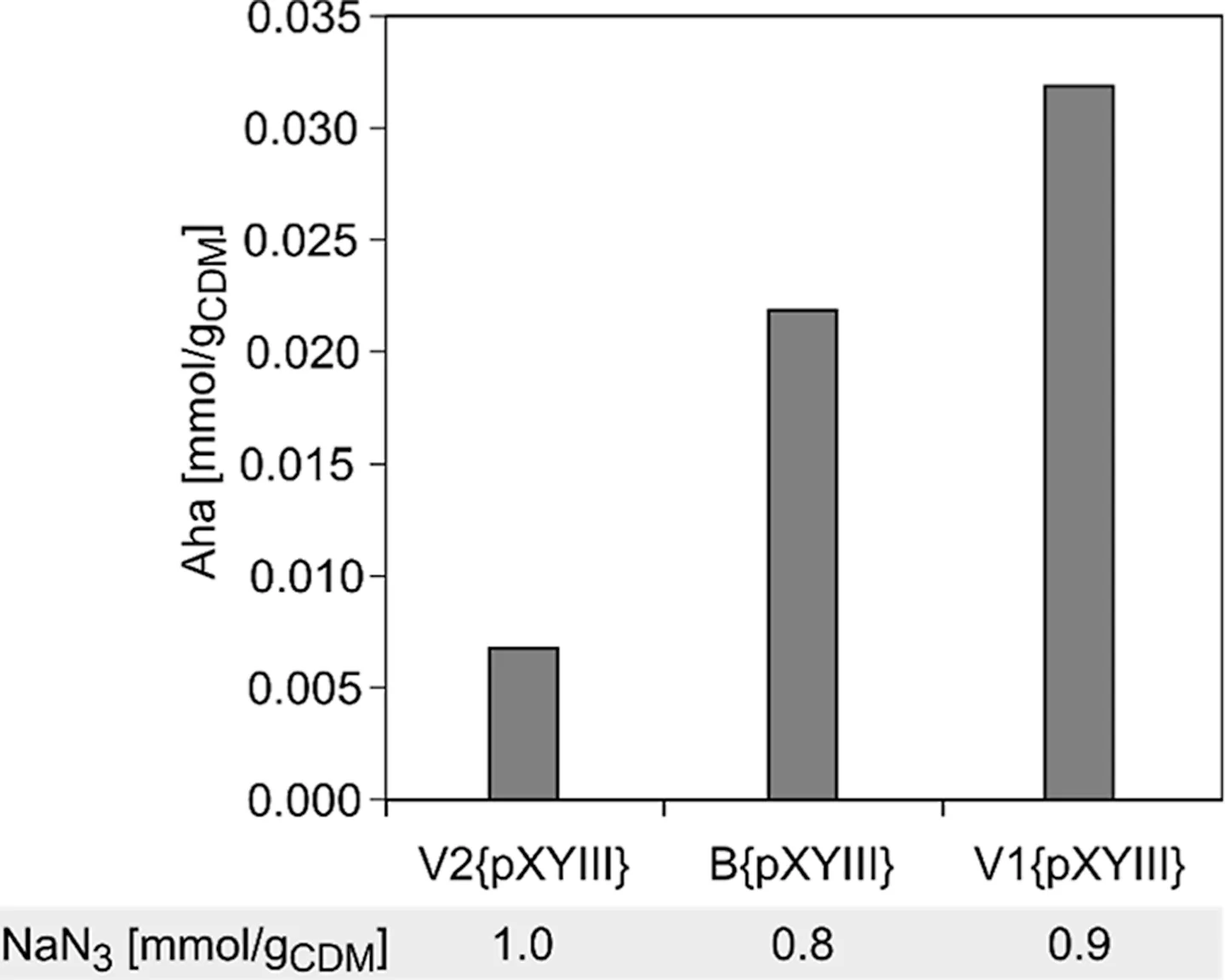
*E. coli* e^X^press V1­{pXYIII} showed highest production of Aha in 1 L fed-batch bioreactor cultures in comparison to the other variants. The Aha concentration in the medium supernatant was assessed using mass spectrometry at 12 h postinduction of *metXY* expression. NaN_3_ was linearly fed after *metXY* induction to reach 30 mmol at the end of fermentation. The resulting mmol NaN_3_/g_CDM_ are indicated for each strain. The details of the bioprocess are outlined in the Materials and Methods section. *E. coli* strain designations are B, BL21­(DE3); V2, e^X^press V2; V1, e^X^press V1. The data from single experiments are shown.

As in the fed-batch-like μ-bioreactor cultures (Figure S1), *metY* was clearly expressed at higher levels than *metX* in fed-batch bioreactor cultures (Figure S5). Figure 6 shows a comparison of the MetXY levels of strain e^X^press V1­{pXYIII} normalized to cell dry mass at different scales, i.e. fed-batch-like μ-bioreactor culture (800 μL, 3.5 g/L CDM) and 1 L fed-batch bioreactor culture (1.2 L, 45 g/L CDM).

**Figure 6:**
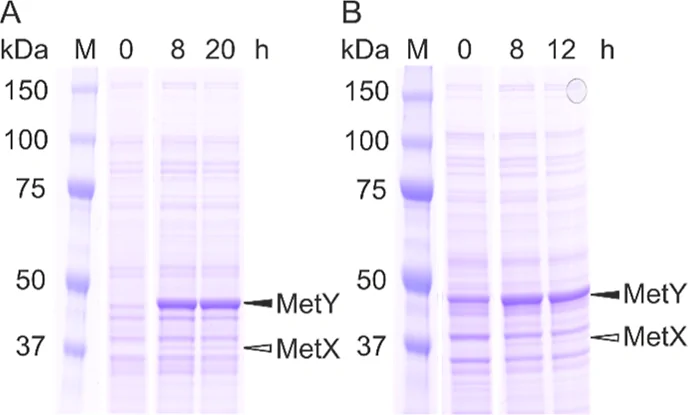
Comparison of *metXY* expression in *E. coli* e^X^press V1­{pXYIII} at different scales. Soluble protein fractions were normalized to 1 mg biomass (CDM). (A) Fed-batch-like μ-bioreactor culture (800 μL, 3.5 g/L CDM); (B) 1 L fed-batch bioreactor culture (1.2 L, 45 g/L CDM). The calculated molecular weights of MetX and MetY are 35 and 47 kDa. A Coomassie-stained 4–12% SDS-PA gel is shown, the sizes of the molecular weight marker bands are indicated on the left margin. The production of MetXY is shown at different times, 8–20 h postinduction (h) of *metXY*. The lanes shown were cropped from the same gel.

There was no detectable difference in *metXY* expression between the two scales. MetX and MetY protein expression levels were respectively 320 mg (10 mg/g_CDM_) and 3.5 g (110 mg/g_CDM_) per liter of e^X^press V1­{pXYIII} fed-batch bioreactor culture (Figure 6B). However, the yield of Aha produced in the fed-batch bioreactor culture was approximately 20-fold lower than in the fed-batch-like μ-bioreactor culture (0.03 mmol/g_CDM_ vs 0.54 mmol/g_CDM_). This difference cannot be attributed to the expression levels of *metXY* genes as they were comparable at the two scales (Figure 6). Currently, we do not have an explanation for this rather unexpected observation. Fed-batch-like μ-bioreactors are useful for screening process parameters at an early development stage,^75^ but their performance metrics do not necessarily translate directly to larger-scale bioreactors. A major difference between the two cultivation scales lies in the oxygen transfer rate (OTR) and utilization under differing biomass concentrations. NaN_3_ is well-known to inhibit components of the respiratory chain in *E. coli*, thereby reducing proton-motive-force-driven ATP synthesis.^76–78^ This effect might be exacerbated in high cell density fed-batch bioreactor cultures where respiratory demand is elevated and respiratory control is tighter. Consequently, energy generation becomes more sensitive to partial inhibition of electron transport even under regulated oxygen conditions. In such cultures, the ATP demand as well as transport and biosynthetic flux might be higher than in fed-batch-like μ-bioreactor cultures. A reduction in cellular energy status can therefore affect pathways involved in amino acid biosynthesis. In particular, a diminished proton motive force impairs membrane-associated transport processes,^59^ including nutrient uptake, which may constrain metabolic flux toward Aha. Taken together, these energy-limiting effects provide a plausible physiological explanation for the reduced Aha yield observed at high cell density in the fed-batch scale relative to the fed-batch-like μ-bioreactor scale. The observed limitation could also arise from differences in MetY turnover between the two scales or variations in the intracellular availability of NaN_3_. Intracellular factors such as metabolic flux distribution through Hse and acetyl-CoA pathways, redox balance, or the pyridoxal-5′-phosphate cofactor availability may influence MetY activity differently under μ-bioreactor conditions in comparison to fed-batch bioreactor cultures. MetY accepts a variety of nucleophiles, such as sulfide, methanethiol, ethanethiol, phenylthiol or cyanide for the synthesis of l-homocysteine, l-methionine, l-ethionine, *S*-phenyl-l-homocysteine and γ-cyano-α-aminobutyric acid.^79–83^ Nevertheless, NaN_3_ appears to be a weak substrate under fed-batch cultivation conditions. The systematic analysis of the bioprocess parameters, alongside the characterization of MetY activity toward NaN_3_ compared to other nucleophiles, will help identify fed-batch bottlenecks and guide further improvement of Aha production via process optimization or enzyme engineering. Ultimately, the engineering of MetY to efficiently accept NaN_3_ as a substrate in combination with an optimized bioprocess that swiftly and quantitatively transforms the azide and OAH to Aha will be key for the scalable simultaneous biosynthesis and incorporation of Aha.

The uptake mechanism of NaN_3_ in *E. coli* is not known yet the compound is highly toxic to the cells,^57,58^ so we focused on the NaN_3_ feeding regime as a key parameter for the Aha biosynthesis bioprocess. In shake-flask cultures, a maximum of 0.8 to 1 mM NaN_3_ can be added to a culture of 3 *D*_600_.^37,59^ The addition of approximately 1 mM NaN_3_ inhibits *E. coli* cell growth on agar plates.^59^ Mutant strains harboring mutations in the SecA protein exhibit a distinctive resilience to the inhibitory effects of NaN_3_, enabling them to sustain growth in the presence of elevated concentrations of NaN_3_, i.e. approximately 4 mM. We hypothesized that the toxicity limit in wild type strains could be avoided by the efficient conversion of NaN_3_ to Aha. To test the hypothesis, we continuously added NaN_3_ resulting in a cumulative addition of 0.6, 0.9, and 1.6 mmol NaN_3_/g_CDM_ to e^X^press V1­{pXYIII} for 12 h postinduction of *metXY* (Figure 7).

**Figure 7:**
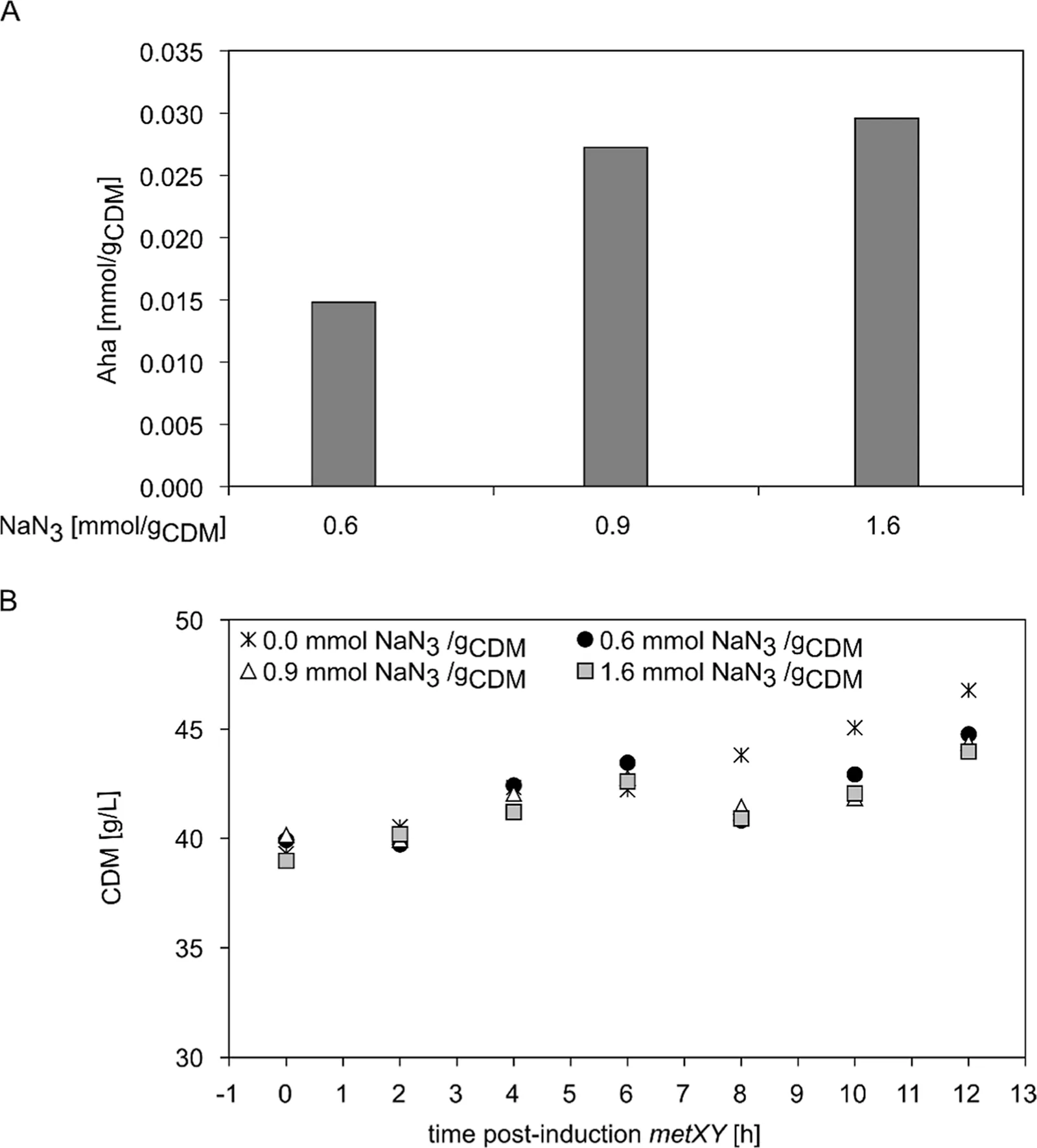
Supplementation of the cells with 1 mmol of NaN_3_/g_CDM_ resulted in the highest Aha production. This supplementation did not have a significant impact on the cell density. The results of 1 L fed-batch bioreactor cultures of *E. coli* e^X^press V1 strain­{pXYIII} are shown. (A) 0.6, 0.9, or 1.6 mmol NaN_3_/g_CDM_ were fed to the three different bioreactor cultures over 12 h postinduction (p.i.) of *metXY*. The specific NaN_3_ supplementations (mmol/g_CDM_) correspond to a total amount of 20, 30, or 50 mmol NaN_3_ fed to the cells until the end of fermentation; (B) NaN_3_ feeding regimes did not influence the CDM of e^X^press V1. 0, 0.6, 0.9, or 1.6 mmol NaN_3_/g_CDM_ were fed to the three different bioreactor cultures over 12 h postinduction of *metXY* as described in the Materials and Methods section. CDM (g/L) values were corrected for dilution effects during the bioprocess. The data from single experiments are shown. n.d., not detected.

The cell density was slightly reduced compared to the control without NaN_3_ but the highest NaN_3_ supplementation of 1.6 mmol NaN_3_/g_CDM_ did not significantly reduce the CDM in comparison to 0.6 mmol NaN_3_/g_CDM_ (Figure 7B). We intended to measure the conversion of NaN_3_ into Aha by HPLC. However, the analysis failed because metabolites and other small molecules released by *E. coli* during the process increased the background compared to the fed-batch-like μ-bioreactor cultures. Alternatively, we determined the Aha levels in the medium supernatant of all fed-batch bioreactor cultures by mass spectrometry. Figure 7 suggests that elevating the intracellular NaN_3_ levels increased the yield of Aha. The maximum Aha yield (0.030 mmol Aha/g_CDM_) was obtained by supplementing the cells with 1.6 mmol NaN_3_/g_CDM_. However, it was not significantly improved in comparison to the supplementation of 0.9 mmol NaN_3_/g_CDM_ (Figure 7A). We measured the remaining NaN_3_ in the culture supernatant at the end of the fermentation. NaN_3_ is converted to ferric azide complex in the presence of a strong acid and ferric trichloride. Tsuge et al. described a spectrophotometric method to measure the ferric azide complex (Figure S6).^84^ Most of the NaN_3_ remained in the culture supernatant, which suggests a rather low conversion efficiency. Respectively, 96%, 83% and 92% of NaN_3_ were recovered in the culture supernatant of the 1 L fed-batch bioreactor cultures that received 0.6, 0.9, and 1.6 mmol NaN_3_/g_CDM_ (Figure S7). Although cell viability was not significantly affected at the highest NaN_3_ supplementation tested (Figure 7B), intermediate supplementation was favorable for Aha biosynthesis. In this context, it might be relevant to further examine and potentially exploit the mutations implicated in the NaN_3_ resistance of some *E. coli* strains.^57–59^ Since an elevated supplementation of NaN_3_ did not lead to a significantly enhanced cellular uptake and conversion of NaN_3_ to Aha, we opted for the specific supplementation of 0.9 mmol NaN_3_/g_CDM_ in the subsequent experiments to maximize the conversion of NaN_3_. An efficient conversion of NaN_3_ to Aha during the bioprocess is key not only to maximize the product yield but also with respect to minimizing the disposal of the toxic educt.

### Bioprocess Optimization Increased the Aha Biosynthesis to Levels Adequate for Supplementation-Based Incorporation

The supplementation of fed-batch-like μ-bioreactor cultures with NH_4_OAc doubled the Aha production as described above. Thus, we scaled up the most suitable specific supplementation of NH_4_OAc according to the CDM of the 1 L fed-batch cultures. Even though the purpose of the fed-batch-like μ-bioreactor cultures was to facilitate the scale-up of the bioprocess, parameters such as the feeding regime, the shear stress, the osmotic pressure or oxygen concentrations differ substantially in the 1 L fed-batch culture. Therefore, we varied the supplementation with NH_4_OAc in the feed medium reaching 5, 10, and 14 mmol NH_4_OAc/g_CDM_ at the end of fermentation. We hypothesized that we could increase the Aha yield by alleviating a potential feedback inhibition of *D. radiodurans* MetX by excess OAH, as previously reported for the enzyme from *C. glutamicum*.^41^ Wei et al. identified a triple F147L-M182I-M240A mutant of MetX from *Leptospira meyeri* that is insensitive to OAH inhibition.^41^ The homologous MetX from *D. radiodurans* contains the corresponding residues L137, I171 and L218. To generate a feedback inhibition-insensitive MetX, we mutated residue L218A while the remaining positions L137 and I171 were not changed. We constructed plasmid pX*YIII, encoding the potentially feedback inhibition-insensitive *metX* variant. The introduction of the feedback inhibition-insensitive MetX tripled the yield of Aha but slightly impaired the cell growth (Figure 8, pXYIII vs pX*YIII). This finding implies that MetX from *D. radiodurans* undergoes feedback inhibition as does the homologous enzyme from *C. glutamicum*.

**Figure 8:**
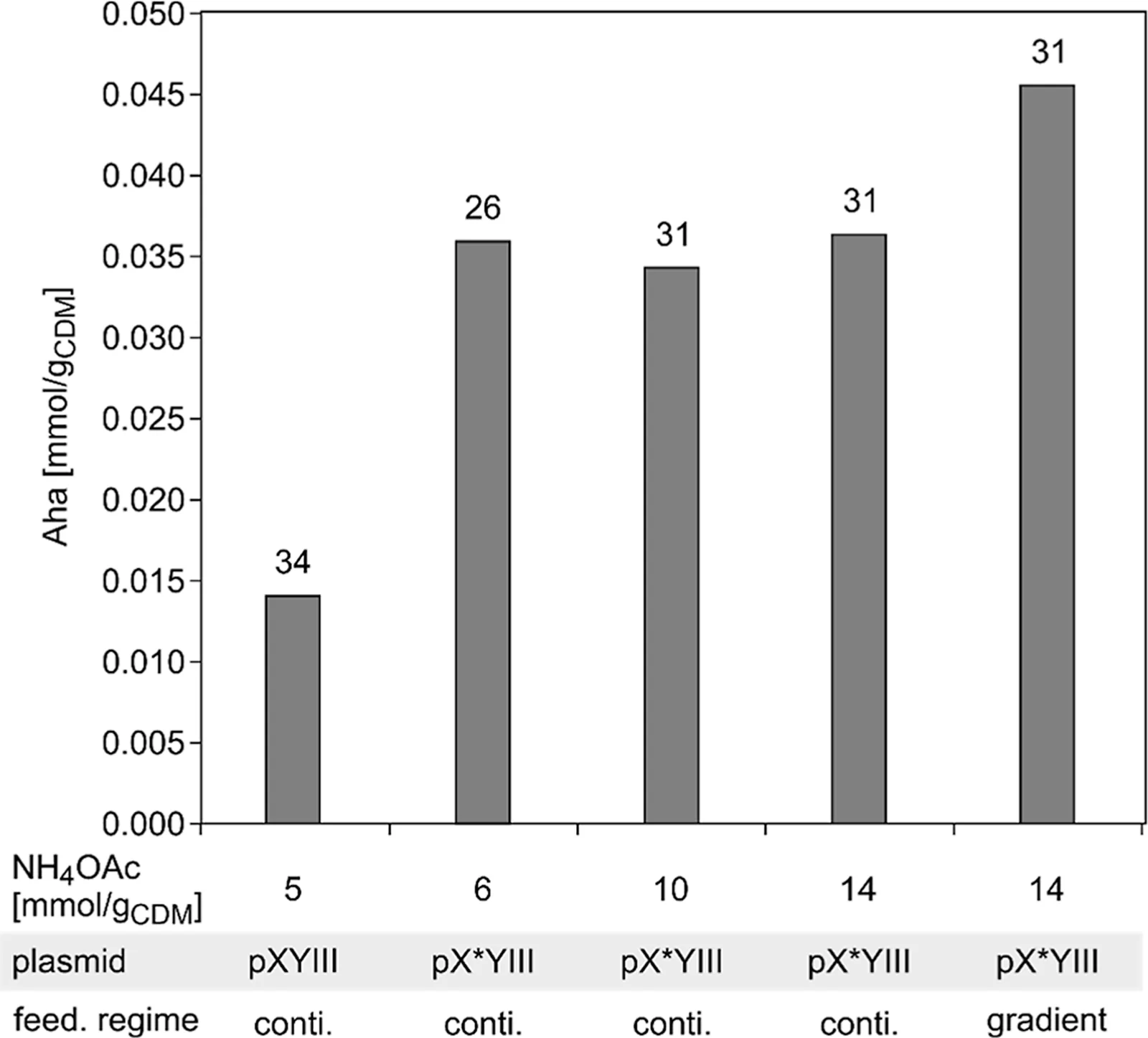
We produced up to 0.05 mmol Aha/g_CDM_ corresponding to a titer of 1.4 mM Aha in 1 L fed-batch bioreactor culture. This increase in Aha biosynthesis was achieved by introducing a feedback inhibition-insensitive *metX* mutant (MetX L218A; X*) into the *E. coli* e^X^press V1 host, the supplementation of 14 mmol NH_4_OAc/g_CDM_ in the feed medium, and a gradient feeding regime of NaN_3_ (feed. regime).^41^ NH_4_OAc was supplemented continuously in the feed medium to reach the indicated specific supplementations at 12 h postinduction (p.i.) of *metXY*. For the continuous (conti.) NaN_3_ feeding regime, a total of 30 mmol NaN_3_ (1 mmol NaN_3_/g_CDM_) was fed over the time span of 12 h. For the gradient NaN_3_ feeding regime, we fed 15 mmol NaN_3_ from 0 to 4 h, 10 mmol from 4 to 8 h and 5 mmol from 8 to 12 h postinduction of *metXY*. The numbers above the bars indicate the measured CDM (g/L) at 12 h after induction of *metXY*. The data from single experiments are shown.

Since the feedback inhibition-insensitive metX allele (X*) increases the consumption of acetyl-CoA,^41^ we hypothesized that the depletion of this central metabolite led to an impairment of the cell growth. We intended to alleviate this impairment by increasing the NH_4_OAc supplementation, which might stimulate intracellular acetyl-CoA regeneration (Figure 8).^71^ Although higher NH_4_OAc levels did not improve Aha yield per biomass, the volumetric Aha titers increased to 0.9, 1.0, and 1.1 mM for 6, 10, and 14 mmol NH_4_OAc/g_CDM_, respectively. This suggests that the higher ammonium acetate supply primarily enhanced biomass formation rather than the specific Aha production capacity of the cells. Finally, we fed the cells with a decreasing gradient of NaN_3_. A total of 30 mmol NaN_3_ was added in three steps, i.e. 15 mmol NaN_3_ from 0 to 4 h, 10 mmol from 4 to 8 h and 5 mmol from 8 to 12 h postinduction of *metXY*. This NaN_3_ feeding regime combined with the addition of 14 mmol NH_4_OAc/g_CDM_ and the feedback inhibition-insensitive MetX mutant yielded a maximal Aha yield of 0.05 mmol/g_CDM_ (1.4 mmol/L). As such, this production level well exceeds the commonly employed Aha concentration of 0.5 mM for SPI in shake-flask cultures as reviewed by Marin et al.^17^ Although we were able to scale-up and monitor the production of Aha in 1 L fed-batch bioreactor cultures for the first time, 1.4 mM Aha might be insufficient for an upscaled SPI process at substantially higher cell density than shake flask cultures. Increased concentrations of NH_4_OAc supplementation in combination with the feedback inhibition-insensitive MetX mutant improved the Aha titer to a certain extent. In contrast, an increase in NaN_3_ did not lead to more Aha (Figure 7A), indicating a bottleneck at the MetY step of the pathway.

Under conditions yielding the highest Aha concentration (1.4 mM) in 1 L fed-batch bioreactor cultures, we investigated whether Aha was unintentionally incorporated into the host proteome (Figure S8). Several factors influence this process. Aha is an approximately 400-fold poorer substrate for MetRS compared to methionine.^16^ In addition, the intracellular methionine concentration, around 100–300 μM,^85^ may be further elevated due to the activity of MetY, which produces the methionine precursor l-homocysteine from *O*-acetylhomoserine and hydrogen sulfide.^83^ Consistent with these considerations, barely detectable fluorescence was observed in the host proteome upon bioorthogonal conjugation with an alkyne fluorophore (Figure S8), indicating negligible misincorporation of biosynthesized Aha. However, elevated Aha production or Met limitation might increase the incorporation levels. To prevent potential cellular effects at higher intracellular Aha levels, *in situ* product removal strategies could be implemented to continuously remove Aha from the culture broth during fermentation. As T7 RNA polymerase, MetX, and MetY contain Met residues, it is relevant to consider producing them prior to NaN_3_ supplementation. This would ensure their synthesis with native Met to avoid reduced activity or stability as reviewed by Marin et al.^17^

To examine whether a prolonged bioprocess would be beneficial, we monitored the accumulation of Aha over time, up to 18 h postinduction of *metXY*. The Aha titer increased during the supplementation of the cells with NaN_3_ and it peaked at 10 h post induction of *metXY*. However, it decreased substantially after we stopped the NaN_3_ feed (Figure 9).

**Figure 9:**
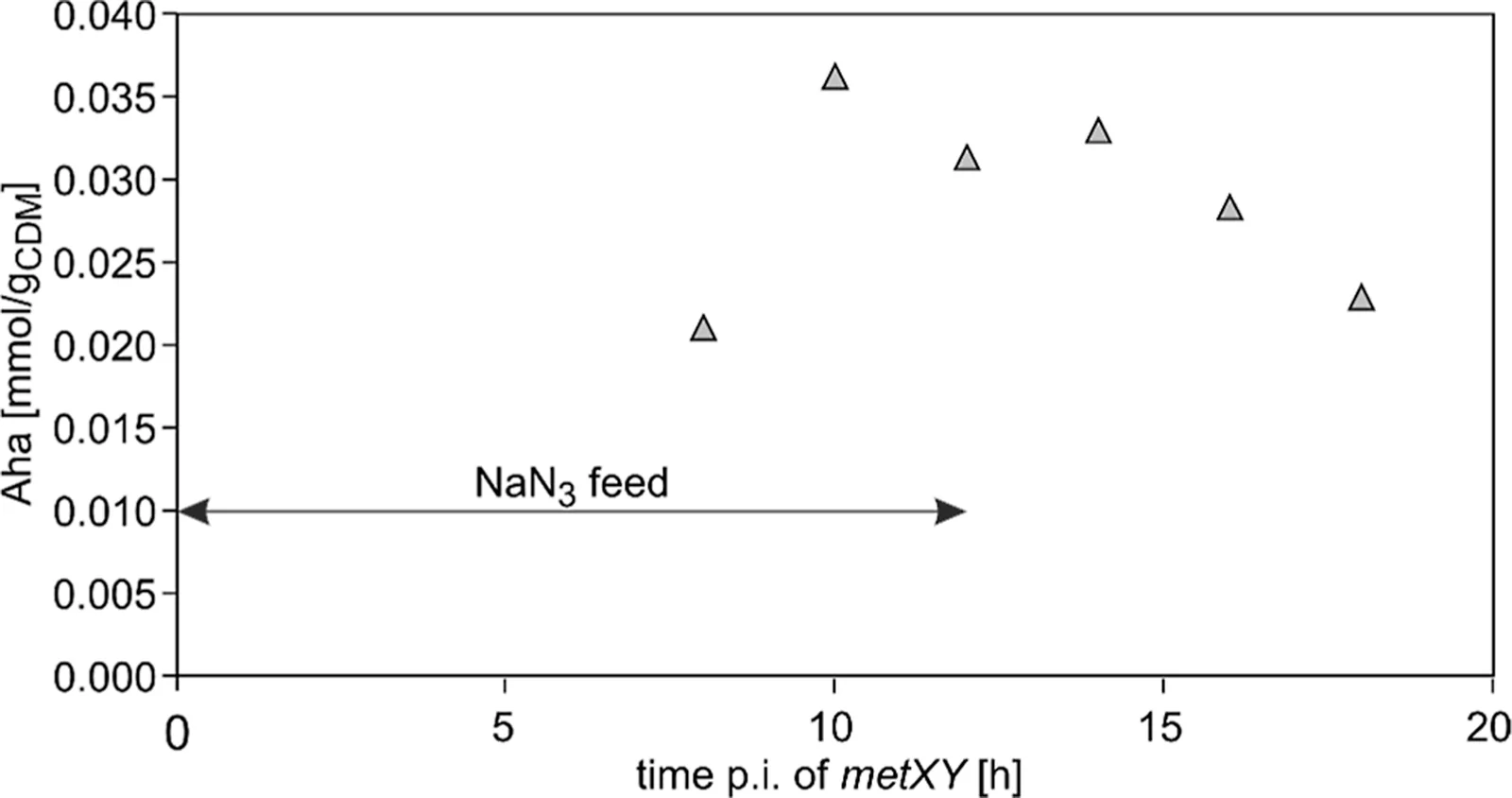
The production of Aha peaked at 10 h postinduction (p.i.) of the *metXY* genes. We monitored the accumulation of Aha during the protein production phase in 1 L fed-batch bioreactor culture. The culture received 30 mmol NaN_3_ (0.9 mmol NaN_3_/g_CDM_) over the time span of 12 h postinduction of *metXY*. NH_4_OAc was continuously supplied through the feed medium to reach a total addition of 180 mmol (6 mmol/g_CDM_) at 18 h postinduction of *metXY*. The concentration of Aha in the culture supernatant of *E. coli* e^X^press V1­{pX*YIII} cells was determined by MS. The data from a single experiment is shown.

Since we considered the volume increase during the fed-batch phase, the observed decrease in Aha levels was not attributable to biomass dilution. Specifically, the Aha yield decreased from 33 to 23 μmol/g_CDM_ between 12 and 18 h postinduction of *metXY* (Figure 9). The e^X^press V1 strain is not auxotrophic for Met and thus the accidental incorporation of Aha into the host proteome, specifically at the level the data in Figure 9 suggests, appears unlikely. Alternatively, the cells might satisfy a potentially increased nitrogen demand by metabolizing the biosynthesized Aha. Rova and co-workers recently highlighted the importance of the nitrogen supply for the microbial production of l-arginine, another nitrogen-rich amino acid.^86^

## Conclusions

For the first time, this study sheds light on the intricacies of Aha biosynthesis in *E. coli*. Employing an e^X^press strain with growth decoupled from target protein production proved efficient in maximizing the expression of the cascade enzymes MetX and MetY. We established various analytical methods to monitor Aha production. Thin-layer chromatography was ideal for fast screening and accurate HPLC protocols were introduced to measure Aha and OAH concentrations. Among the fed-batch-like μ-bioreactor cultures, we found that the highest yield was obtained with *E. coli* eXpress V1­{pXYIII}. This result correlated with the strongest *metX* expression in this strain as observed in the SDS-PAGE analysis. Aha’s biotechnological production was upscaled to 1 L fed-batch bioreactor cultures. The complexity of the culture medium challenged the determination of Aha by HPLC due to the high background, which we were able to eliminate by a HPLC-MS approach. We observed a drop in Aha yield between the fed-batch-like μ-bioreactor culture and the fed-batch bioreactor culture. This difference might be attributed to specific constraints in the fed-batch bioreactor culture; while OTR is a critical parameter for high-cell-density cultivation, it remains uncontrolled at the fed-batch-like μ-bioreactor scale. Additionally, the NaN_3_ levels in the culture supernatant were measured spectrophotometrically. Fine-tuning of the NaN_3_ feeding regime and of the production time improved the conversion of NaN_3_ to Aha. Our optimization efforts included the introduction of an OAH feedback inhibition-insensitive variant of MetX. The analysis of the metabolites in fed-batch-like μ-bioreactor cultures revealed that NH_4_OAc supplementation doubled the biosynthesis of the intermediary OAH. We achieved a maximal Aha production of 1.4 mM or 0.05 mmol/g_CDM_ in 1 L fed-batch bioreactor cultures of *E. coli* e^X^press V1 expressing the feedback inhibition-insensitive *metX* together with *metY* in a tandem array; supplementation with 430 mM NH_4_OAc; and a gradient feeding regime for NaN_3_ (Figure 8). Our study contributes to advancing Aha to biotechnological applications. We observed that the scale-up from the fed-batch-like μ-bioreactor to the fed-batch bioreactor cultures resulted in a markedly different Aha yield. Our findings suggest that MetY may function as the rate-limiting enzyme in the Aha biosynthesis pathway. More research is necessary to substantially improve the conversion of the toxic precursor NaN_3_ such that the scalable cobiosynthesis of Aha and its direct incorporation into a protein of interest become viable. Future work should focus on improving NaN_3_ conversion efficiency through protein engineering of MetY, for example via rational design and directed evolution, combined with the implementation of azide-tolerant production strains, to enable scalable biosynthesis and cotranslational incorporation of Aha.

## Supplementary Material



## Notes

Additional experimental method; SDS-PA gels of *metXY* expression at different scales (Figures S1 and S5) and Aha misincorporation into the host proteome (Figure S8); biological replicates (Figures S2 and S3); thin-layer chromatography to monitor Aha biosynthesis (Figure S4); colorimetric assay for azide detection (Figure S6); residual NaN_3_ in media supernatants (Figure S7); primer list (Table S1), DNA and amino acid sequences list (Table S2), media recipes (Tables S3–S5); strains and plasmids used in the study (Table S6) (PDF)

## List of Abbreviations

Aha: l-azidohomoalanine
CDM: cell dry mass
*D*_600_: attenuance at 600 nm
DO: dissolved oxygen
Hse: l-homoserine
IPTG: isopropyl β-d-1-thiogalactopyranoside
Met: l-methionine
MetX: l-homoserine-*O*-acetyltransferase
MetY: *O*-acetyl-l-homoserine sulfhydrylase
MS: mass spectrometry
NaN_3_: sodium azide
ncAA: noncanonical amino acid
NH_4_OAc: ammonium acetate
OAH: *O*-acetyl-l-homoserine
OPA-HPLC: *o*-phthalaldehyde derivatization combined with high-performance liquid chromatography
OTR: oxygen transfer rate
RNAP: RNA polymerase
SDS-PAGE: sodium dodecyl sulfate-polyacrylamide gel electrophoresis
SPI: supplementation-based incorporation
TLC: thin-layer chromatography
