# Supplementary material for: Metabolic Engineering and Bioprocess Optimization Strategies Promote l‑Azidohomoalanine Biosynthesis in Fed-Batch Bioreactor Culture
Source: ACS Omega. 2026 Sep 29;11(40):61208–20. doi: 10.1021/acsomega.6c05952 (PMC13670341; doi:10.1021/acsomega.6c05952)
Supplement: Supplementary file 1 [file ao6c05952_si_001.pdf]

Supporting Information

Metabolic engineering and bioprocess optimization strategies promote L-azidohomoalanine biosynthesis in fed-batch bioreactor culture

Claudia Lacombe[1], Gerald Striedner[1] and Birgit Wiltschi[1,2]*

[1] BOKU University, Department of Biotechnology and Food Sciences, Institute for Bioprocess Science and Engineering, Vienna, Austria

[2] acib - Austrian Centre of Industrial Biotechnology, Vienna, Austria

*Correspondence:

Birgit Wiltschi

birgit.wiltschi@boku.ac.at

Supporting Methods

Assessment of Aha misincorporation into the host proteome

To assess incorporation of biosynthesized Aha into cellular proteins, strain promoted azide-alkyne cycloaddition was performed using DBCO-AZDye546 (Jena Bioscience, Jena, Germany). DBCO-AZDye546 was prepared as a 10 mM stock solution in DMSO and added to cell lysates at a final concentration of 1 mM. Labeling reactions were incubated for 1 h at 22 °C with shaking at 600 rpm. Following labeling, 30 µL of each reaction was loaded onto 8% SDS-PA gels and analyzed by fluorescence imaging using a Bio-Rad fluorescence imager (Bio-Rad Laboratories, Hercules, USA). Gels were subsequently stained with Coomassie Brilliant Blue to visualize total protein. Fluorescence labeling of Aha-producing samples was compared with labeling in the absence of biosynthesized Aha (cells cultured without NaN_3) to assess Aha incorporation into the cellular proteome.

Supporting Figures

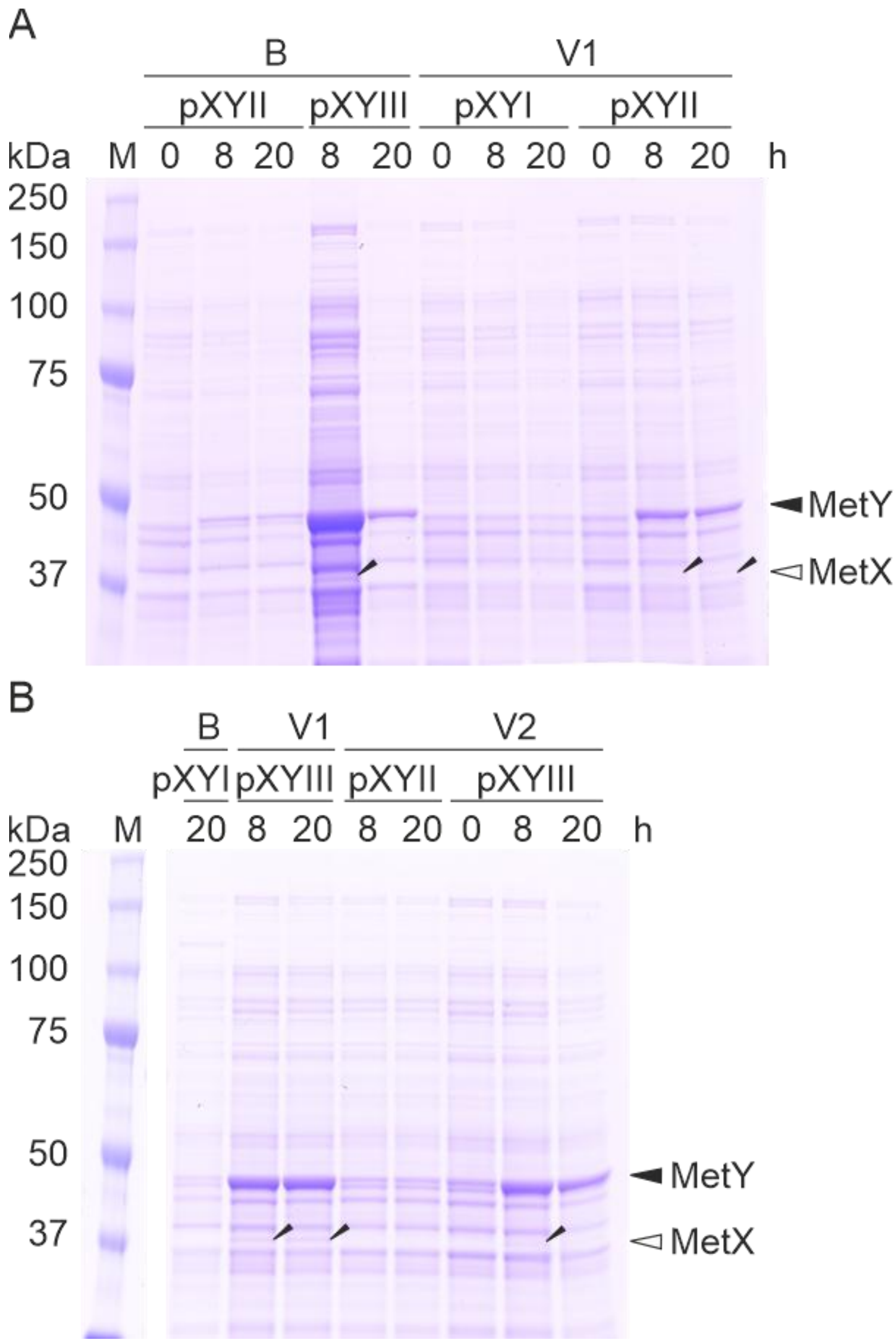

Figure S1. Strain e^{X}press V1{pXYIII} showed the highest production of MetX and MetY in µ-bioreactor culture. Soluble protein fractions were analyzed by SDS-PAGE 0, 8 and 20 h into induction of *metXY*. (A) BL21(DE3){pXYII}, BL21(DE3){pXYIII}, e^{X}press V1{pXYI}, e^{X}press V1{pXYII}; (B) BL21(DE3){pXYI}, e^{X}press V1{pXYIII} and e^{X}press V2{pXYII}, e^{X}press V2{pXYIII}. The architectures of plasmids pXYI-III are shown in Figure 2. Protein bands corresponding to MetX (calculated molecular weight (MW_{calc}), 35 kDa) and MetY (MW_{calc} 47 kDa) are labeled by white and black arrows. h, hours post-induction of *metXY* genes; M, molecular weight marker, the size of the marker bands is indicated on the left

margin of the gel image. Coomassie stained 8% SDS-PA gels are shown. In panel A, the BL21(DE3){pXYIII}, 8 h sample contained a higher total protein load than the other lanes.

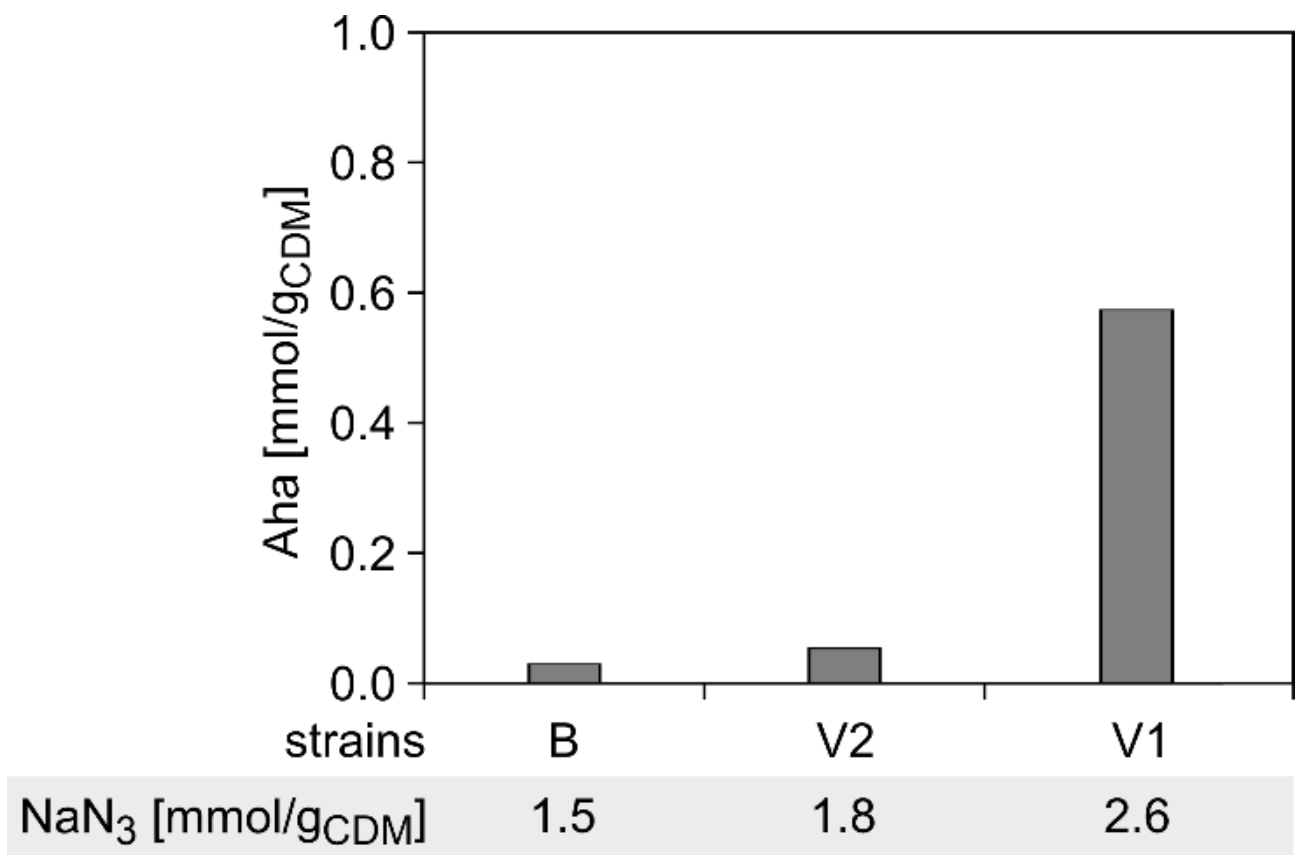

Figure S2. Biological replicate of the experiment shown in Figure 3. Biological replicates consisted of independent microbioreactor cultivations initiated from separate pre-cultures of the same strain and grown under identical conditions. The Aha concentration in the culture supernatant was determined by HPLC as described in the Materials and Methods section at the end of the fermentation. All strains carried plasmid pXYIII for the expression of *metXY*. Strain designations are B, BL21(DE3); V2, e^{X}press V2; V1, e^{X}press V1. At induction of *metXY*, NaN_3 was added in pulses every hour to reach a total addition of 5 µmol. The mmol NaN_3 /g_{CDM} varied because the cultures reached different final cell densities.

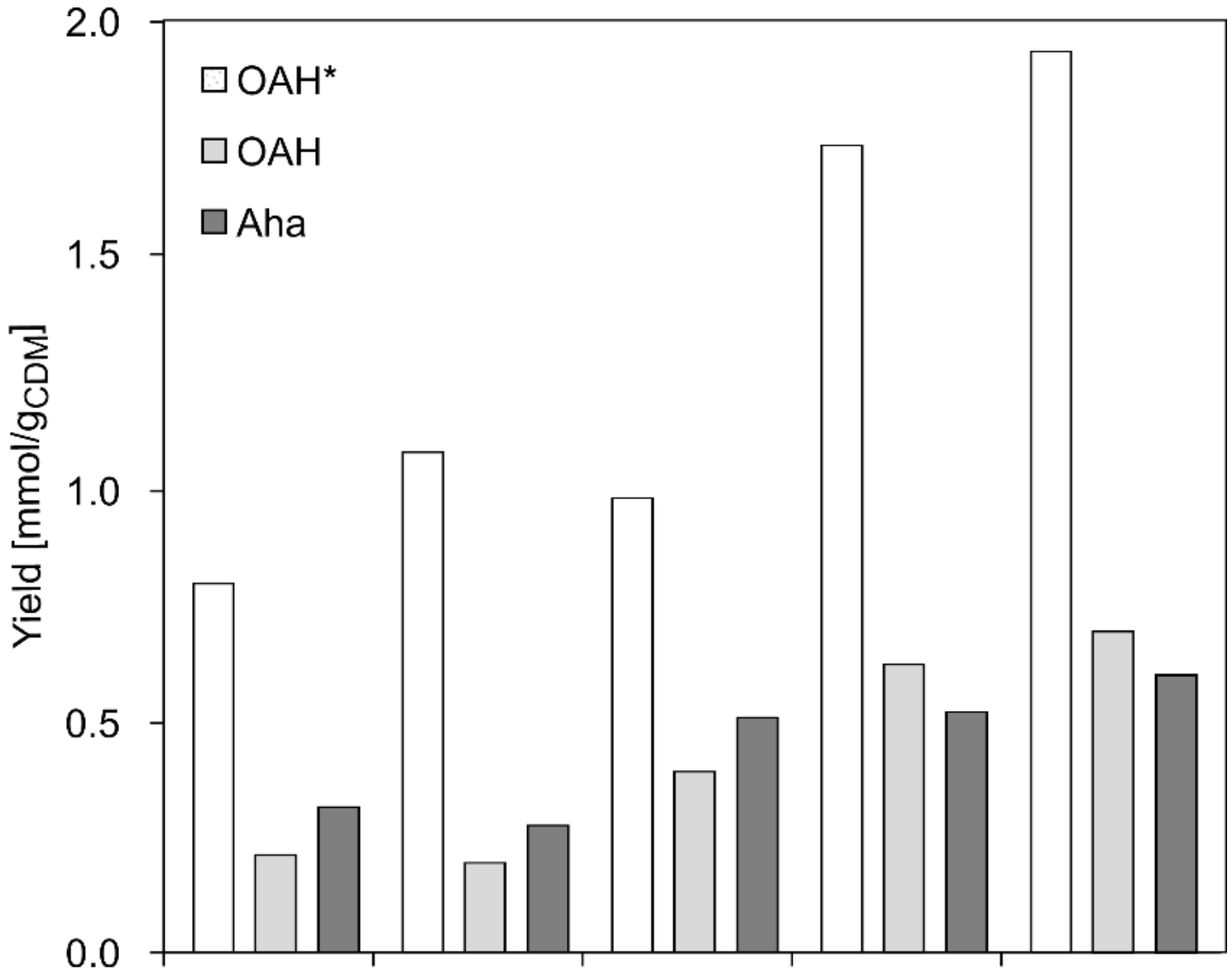

NaN_3 [mmol/g_{CDM}]	-	1.7	1.7	-	1.8	1.8	-	1.7	1.7	-	2.1	2.1	-	2.2	2.2
NH_4OAc [mmol/g_{CDM}]	-	-	-	-	-	-	-	-	-	9	10	10	18	22	22
NaCit [mmol/g_{CDM}]	-	-	-	6	7	7	-	-	-	-	-	-	-	-	-
Hse [mmol/g_{CDM}]	-	-	-	-	-	-	1	1	1	-	-	-	-	-	-

Figure S3. Biological replicate of the experiment shown in Figure 4. Biological replicates consisted of independent microbioreactor cultivations initiated from separate pre-cultures of the same strain and grown under identical conditions. The impact of different media additives on the production of Aha and its intermediary metabolite OAH were analyzed in fed-batch-like µ-bioreactor cultures of *E. coli* e^{X}press V1 {pXYIII} 8 h post-induction of *metXY* expression. The concentration of the intermediary metabolite OAH was measured with and without (OAH*) the addition of 5 µmol NaN_3. Refer to the Materials and Methods section for details of HPLC analysis of Aha and OAH. At induction of *metXY*, NaN_3 was added in pulses every hour to reach a total addition of 5 µmol equaling 2 mmol/g_{CDM}.

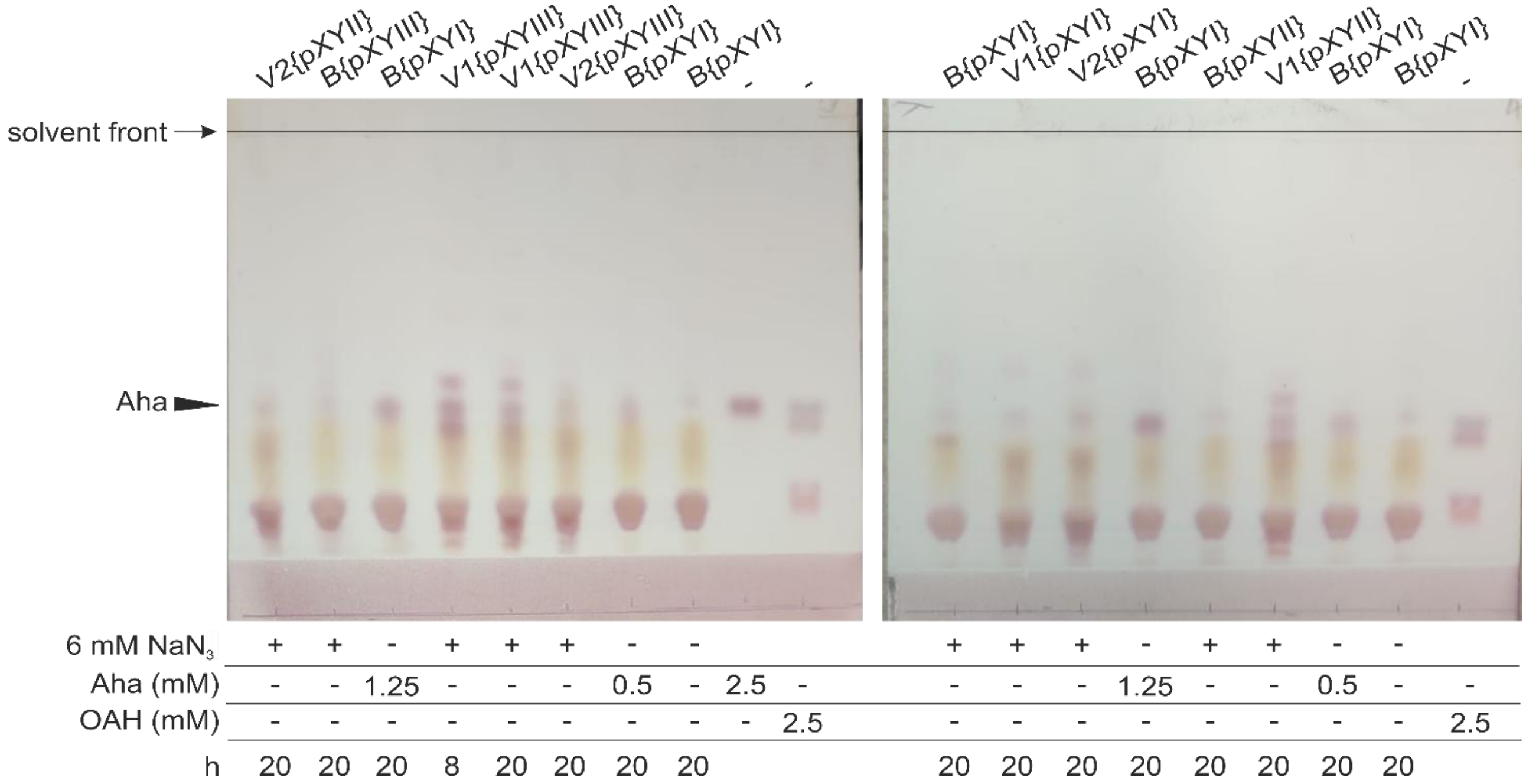

Figure S4. Thin-layer chromatography allows fast screening of Aha producing strains. Aha was biosynthesized in fed-batch like µ-bioreactor cultures supplemented with 6 mM NaN_3 in total. Samples were spiked with Aha or OAH as positive controls where indicated. BL21(DE3){pXYI}, which did not receive NaN_3, was used as a negative control. The Aha spot is indicated by a black arrow. Strain designations are B; *E. coli* BL21(DE3), V2; *E. coli* e^{X}press V2, V1; *E. coli* e^{X}press V1. Refer to Figure 2 for plasmid designations. Thin-layer silica plates were developed in $CHCl_3$/96% EtOH/CH_3COOH/25% NH_3 at a ratio of 5:2.6:0.9:0.6 (% v/v) and stained with ninhydrin as described in the Materials and Methods section of the main text.

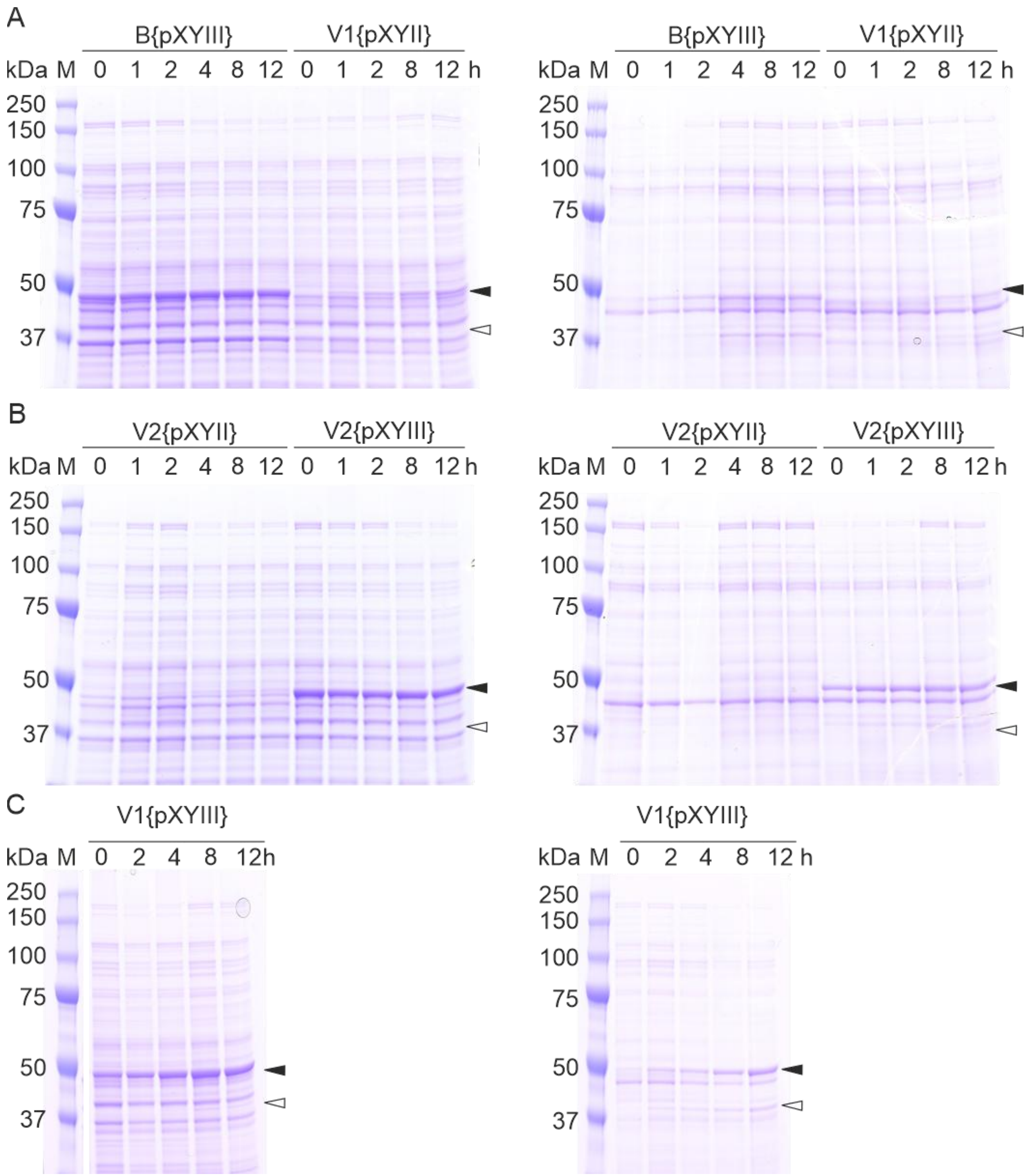

Figure S5. Strain e^{X}press V1{pXYIII} showed the highest production of MetX and MetY 1 L fed-batch bioreactor cultures. Proteins were analyzed by SDS-PAGE 0-12 h into induction of *metXY*. Soluble and insoluble protein fractions are shown on the left and right SDS-PA gels, respectively. (A) BL21(DE3){pXYIII} and e^{X}press V1{pXYII}; (B) e^{X}press V2{pXYII} and e^{X}press V2{pXYIII}; (C) e^{X}press V1{pXYIII}. The architectures of plasmids pXYI-III are

shown in Figure 2. Protein bands corresponding to MetX (MW_{calc} 35 kDa) and MetY (MW_{calc} 47 kDa) are highlighted by white and black arrows. h, hours post-induction of *metXY* genes; M, molecular weight marker, the size of the marker bands is indicated on the left margin of the gel image. Coomassie stained 8% SDS-PA gels are shown.

A

$$NaN_3 \text{ in 10 mM in HCl} \rightarrow HN_3$$
$$Fe^{+3} + HN_3 \rightarrow FeN_3{}^{2+} + H^+$$

B

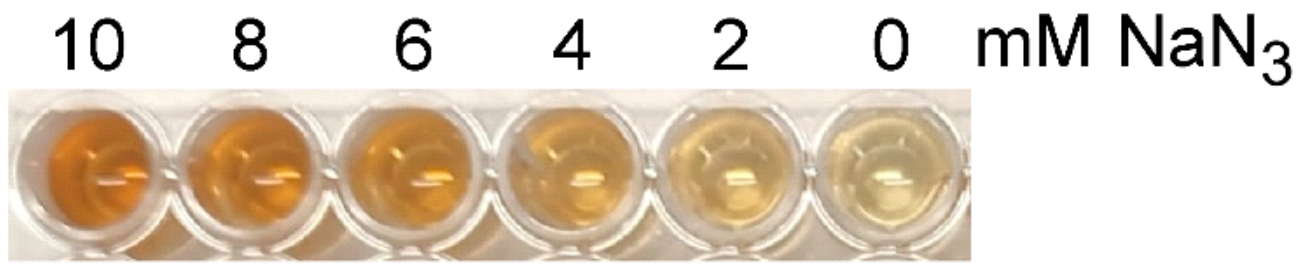

Figure S6. NaN_3 was measured by spectrophotometric assessment of ferric azide complex formation. (A) Ferric azide complex formation. In the presence of a strong acid, NaN_3 is converted to hydrazoic acid (HN_3). The hydrazoic acid reacts with ferric ion to form a colored ferric azide complex (maximum absorbance at 460 nm); (B) 0 to 10 mM NaN_3 were used as calibration standards.

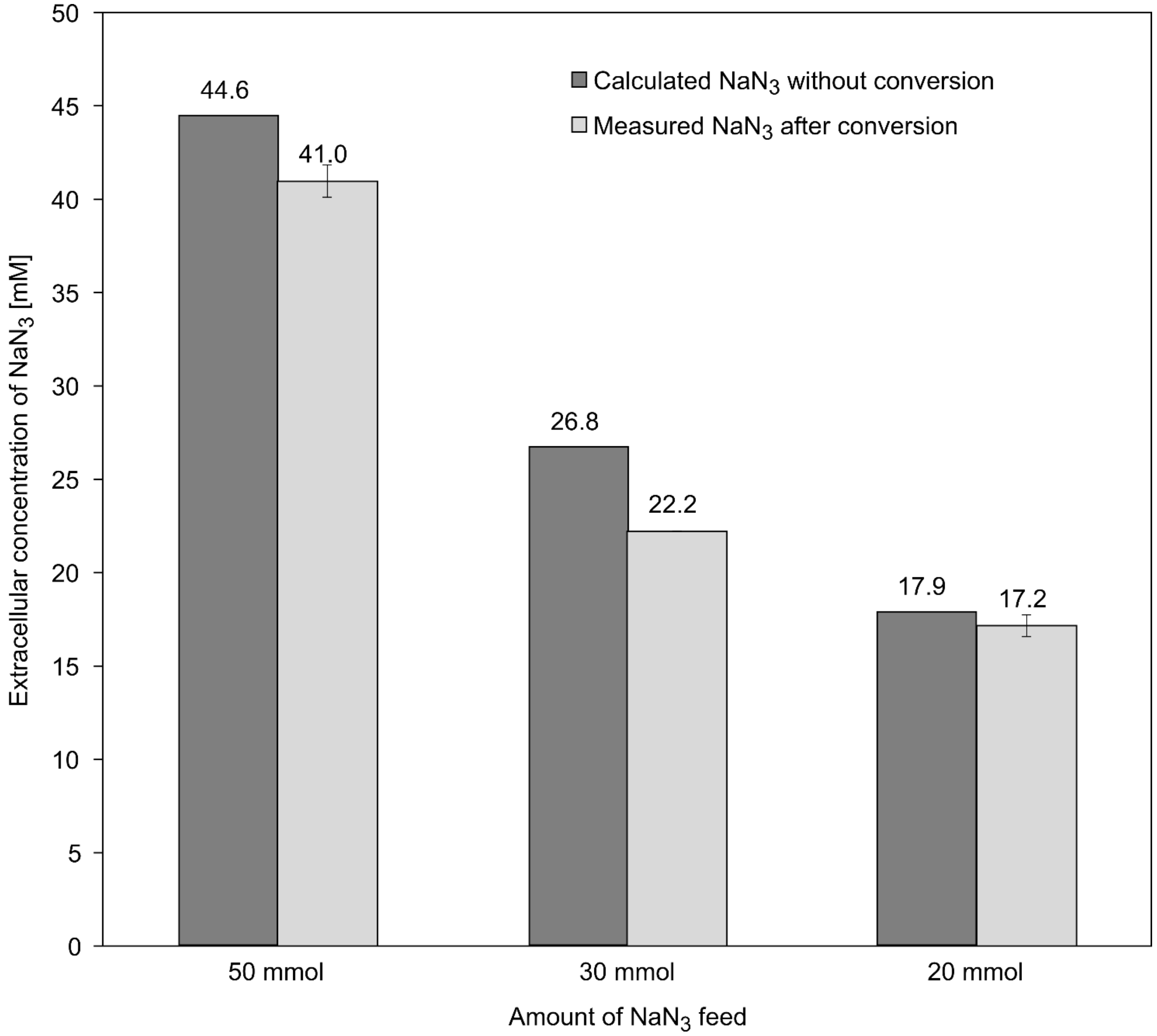

Figure S7. The residual NaN_3 concentration was determined in the medium of 1 L fed-batch bioreactor cultures. Dark grey bars indicate the theoretical NaN_3 concentration if the fed NaN_3 was not consumed by the cells or converted to Aha. Light grey bars indicate the measured residual NaN_3 concentration in the medium. Numbers above the bars indicate the depicted values and the error bars represent the standard deviation of three technical replicates.

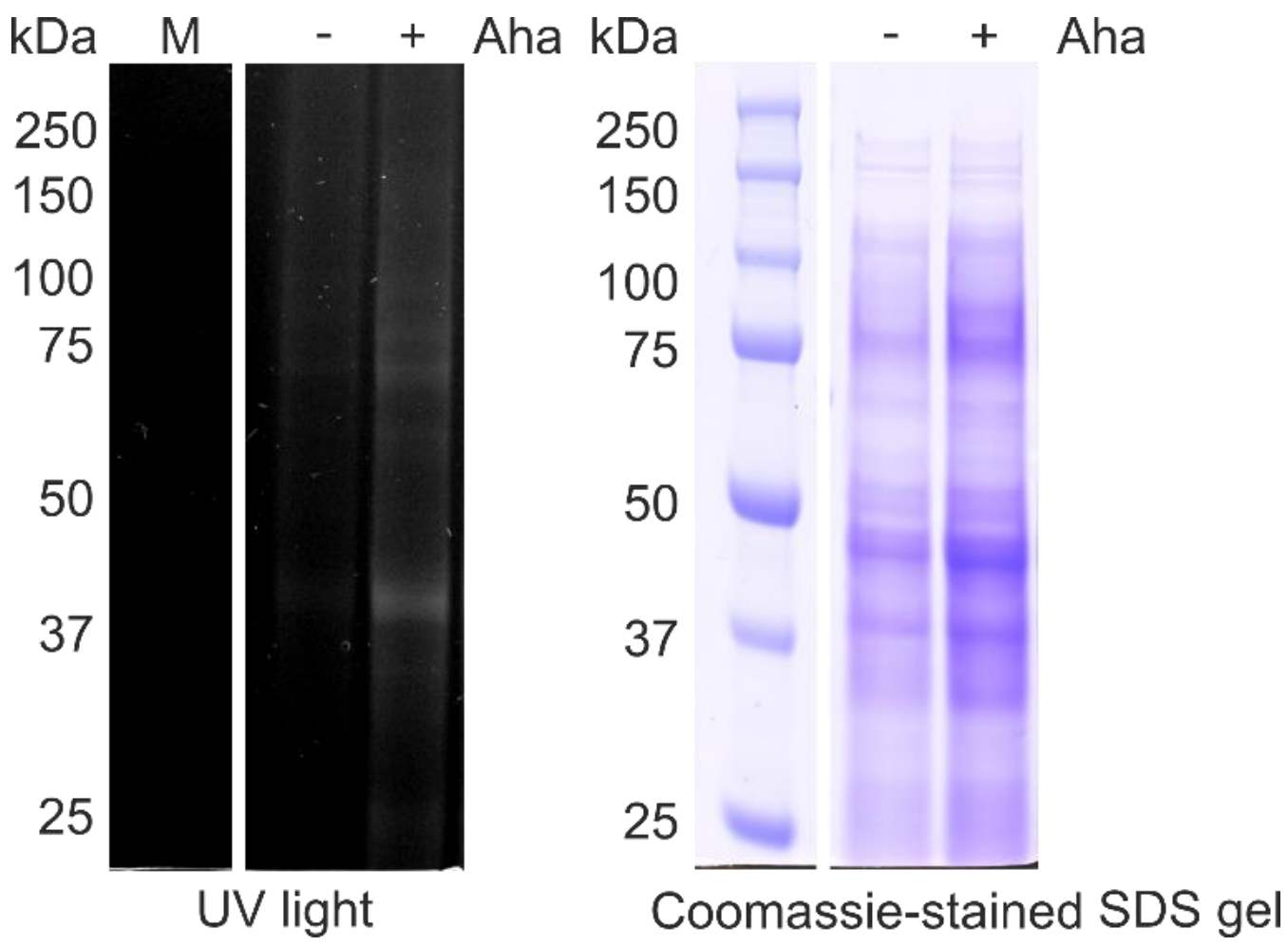

Figure S8. No substantial misincorporation of Aha into the proteome was detected during its biosynthesis in 1 L fed-batch bioreactor culture. The culture exhibiting the highest Aha biosynthesis (Figure 8), accumulating up to 0.05 mmol Aha /g_{CDM} (1.4 mM), was used for proteome labeling with DBCO-AZDye546. Incorporation of Aha into the cellular proteome was assessed as described in the Supporting Methods section. Briefly, we labeled azide-containing proteins with DBCO-AZDye546 using strain-promoted azide-alkyne cycloaddition, followed by SDS-PAGE and fluorescence imaging. Labeling reactions were performed either in the presence or absence (without NaN_3) of biosynthesized Aha. Incorporation of Aha into the proteome was assessed at 12 h post-induction of *metXY*. Proteins were separated on 8% SDS-PA gels and visualized by fluorescence imaging (left) and Coomassie staining (right). h, hours post-induction of metXY expression; M, molecular weight marker, band sizes indicated on the left margin of the gel.

Supporting Tables

Table S1. Primers and templates for the construction of the expression vectors used in this work. BsaI-HFv2 restriction sites are indicated in bold.

PCR product	Primer 1	Primer 2	Template
bsa_lacIq_bsa	GATTAC**GGTCTC**AAGGAGATCATAAAAAACGC	TTCTAC**GGTCTC**AAGCCAGAGCGCTATGCATG	bsa_lacIq_MCS
bsa_cer_kanR_bsa	CATTATAC**GGTCTC**CTCAGGCGC	TGATCAAC**GGTCTC**GTCCGATC	bsa_cer_kanR_bsa
bsa_ori_bom_rop_bsa	ATGATTAC**GGTCTC**GCGGAGC	GCATAC**GGTCTC**ATCCTGTCTTTAAACG	bsa_ori_bom_rop_bsa
bsa_T7p_MetY_RBS_bsa_pXYI	GGCTAC**GGTCTC**AGGCTGACGAGGATATCGGCTATCCTAATACGAC	CTAGTGA**GGTCTC**TATCTCCTTCTTAAAGTTAAACAAACTGACCTTACTTAACTCCAACGGTTTGGCTCTG	BSmetY
bsa_MetX_T7t_bsa_pXYI	AAGGAGA**GGTCTC**TAGATATGAGAATGACCGCAGTTCTGGCAGGTC	GGCTAC**GGTCTC**CCTGAGCCAACTTCGTCCATGATGCTGTCAGTGG	DRmetX
bsa_T7p_MetY_T7t_bsa_pXYII	GGCTAC**GGTCTC**CCTGAGCCAATGGATCCAAGCAGTTCAAAAAACCC	AAGGAGA**GGTCTC**TACGAGACGAGGATATCGGCTATCCTAATACGAC	BSmetY
bsa_T7p_MetX_T7t_bsa_pXYII	GGCTAC**GGTCTC**ATCGTCGATCCCGCGAAATTAATACGACTC	GGCTAC**GGTCTC**AGGCTCTTCGTCCATGATGCTGTCAGTGG	DRmetX
bsa_T7p_MetX_T7t_bsa_pXYIII	GGCTAC**GGTCTC**ATCGTCTTCGTCCATGATGCTGTCAGTGG	GGCTAC**GGTCTC**AGGCTCGATCCCGCGAAATTAATACGACTC	DRmetX
bsa_T7p_metY_T7t_bsa_pXYIII	GGCTAC**GGTCTC**CCTGAGCCAATGGATCCAAGCAGTTCAAAAAACCCC	AAGGAGA**GGTCTC**TACGAGACGAGGATATCGGCTATCCTAATACGAC	BSmetY

Table S2. DNA and amino acid sequences used for the production of pXYI, pXYII, pXYIII and pX*YIII expression constructs. BsaI-HFv2 restriction sites are indicated in bold.

Name	DNA sequence (5'→3')
bsa_cer_kanR_bsa	**GGTCTC**CTCAGGCGCTAGAATTCAGGAACTATATCCGGTGGCGAATGGGACGCGCCCTGTAGCGGCGCATTAAGC<br>GCGGAATACAGGAACGCACGCTGGATGGCCCTTCGCTGGGATGGTGAAACCATGAAAAATGGCAGCTTCAGTGGA<br>TTAAGTGGGGGTAATGTGGCCTGTACCCTCTGGTTGCATAGGTATTCATACGGTTAAAATTTATCAGGCGCGATC<br>GCGGCAGTTTTTCGGGTGGTTTGTTGCCATTTTTACCTGTCTGCTGCCGTGATCGCGCTGAACGCGTTTTAGCGG<br>TGCGTACAATTAAGGGATTATGGTAAATCCACTTACTGTCTGCCCTCGTAGCCATATTTTAACAAAATATTAACG<br>TTTACAATTTCAGGTGGCACTTTTCGGGGAAATGTGCGCGGAACCCCTATTTGTTTATTTTTCTAAATACATTCA<br>AATATGTATCCAAAAGGCCATCCGTCAGGATGGCCTTCTTTAGAAAAACTCATCGAGCATCAAATGAAACTGCAA<br>TTTATTCATATCAGGATTATCAATACCATATTTTTGAAAAAGCCGTTTCTGTAATGAAGGAGAAAACTCACCGAG<br>GCAGTTCCATAGGATGGCAAGATCCTGGTATCGGTCTGCGATTCCGACTCGTCCAACATCAATACAACCTATTAA<br>TTTCCCCTCGTCAAAAATAAGGTTATCAAGTGAGAAATCACCATGAGTGACGACTGAATCCGGTGAGAATGGCAA<br>AAGTTTATGCATTTCTTTCCAGACTTGTTCAACAGGCCAGCCATTACGCTCGTCATCAAAATCACTCGCATCAAC<br>CAAACCGTTATTCATTCGTGATTGCGCCTGAGCGAGACGAAATACGCGATCGCTGTTAAAAGGACAATTACAAAC<br>AGGAATCGAATGCAACCGGCGCAGGAACACTGCCAGCGCATCAACAATATTTTCACCTGAATCAGGATATTCTTC<br>TAATACCTGGAATGCTGTTTTACCGGGGATCGCAGTGGTGAGTAACCATGCATCATCAGGAGTACGGATAAAATG<br>CTTGATGGTCGGAAGAGGCATAAATTCCGTCAGCCAGTTTAGTCTGACCATCTCATCTGTAACATCATTGGCAAC<br>GCTACCTTTGCCATGTTTCAGAAACAACTCTGGCGCATCGGGCTTCCCATACAATCGATAGATTGTCGCACCTGA<br>TTGCCCGACATTATCGCGAGCCCATTTATACCCATATAAATCAGCATCCATGTTGGAATTTAATCGCGGCCTAGA<br>GCAAGACGTTTCCCGTTGAATATGGCTCATGCGAAACGATCCTCATCCTGTCTCTTGATCAGATCTTGATCCCCT<br>GCGCCATCAGATCCTTGGCGGCAAGAAAGCCATCCAGTTTACTTTGCAGGGCTTCCCAACCTTACCAGAGGGCGC<br>CCCAGCTGGCAATTCCGGAAAAGCTTATACATCCCCGTAGAAAAGATCGGAC**GAGACC**
bsa_lacIq_MCS	AC**GGTCTC**AAGGAGATCATAAAAAACGCCCGGCGGCAACCGAGCGTTCTGAATCACTGCCCGCTTTCCAGTCGGG<br>AAACCTGTCGTGCCAGCTGCATTAATGAATCGGCCAACGCGCGGGGAGAGGCGGTTTGCGTATTGGGCGCCAGGG<br>TGGTTTTTCTTTTCACCAGTGAGACGGGCAACAGCTGATTGCCCTTCACCGCCTGGCCCTGAGAGAGTTGCAGCA<br>AGCGGTCCACGCTGGTTTGCCCCAGCAGGCGAAAATCCTGTTTGATGGTGGTTAACGGCGGGATATAACATGAGC<br>TGTCCTCGGTATCGTCGTATCCCACTACCGAGATATCCGCACCAACGCGCAGCCCGGACTCGGTAATGGCGCGCA<br>TTGCGCCCAGCGCCATCTGATCGTTGGCAACCAGCATCGCAGTGGGAACGATGCCCTCATTCAGCATTTGCATGG<br>TTTGTTGAAAACCGGACATGGCACTCCAGTCGCCTTCCCGTTCCGCTATCGGCTGAATTTGATTGCGAGTGAGAT<br>ATTTATGCCAGCCAGCCAGACGCAGACGCGCCGAGACAGAACTTAATGGGCCCGCTAACAGCGCGATTTGCTGAT<br>GACCCAATGCGACCAGATGCTCCACGCCCAGTCGCGTACCGTCCTCATGGGAGAAAATAATACTGTTGATGGGTG<br>TCTGGTCAGAGACATCAAGAAATAACGCCGGAACATTAGTGCAGGCAGCTTCCACAGCAATGGCATCCTGGTCAT<br>CCAGCGGATAGTTAATGATCAGCCCACTGACGCGTTGCGCGAGAAGATTGTGCACCGCCGCTTTACAGGCTTCGA<br>CGCCGCTTCGTTCTACCATCGACACCACCACGCTGGCACCCAGTTGATCGGCGCGAGATTTAATCGCCGCGACAA<br>TTTGCGACGGCGCGTGCAGGGCCAGACTGGAGGTGGCAACGCCAATCAGCAACGACTGTTTGCCCGCCAGTTGTT<br>GTGCCACGCGGTTGGGAATGTAATTCAGCTCCGCCATCGCCGCTTCCACTTTTTCCCGCGTTTTCGCAGAAACGT<br>GGCTGGCCTGGTTCACCACGCGGGAAACGGTCTGATAAGAGACACCGGCATACTCTGCGACATCGTATAACGTTA<br>CTGGTTTCACATTCACCACCCTGAATTGACTCTCTTCCGGGCGCTATCATGCCATACCGCGAAAGGTTTTGCACC<br>ATTCGATGGTGTCGCATGCATAGCGCTCTGGCTTG
bsa_ori_bom_rop_bsa	**GGTCTC**GCGGAGCTGCCCGTAGAAAAGATCAAAGGATCTTCTTGAGATCCTTTTTTTCTGCGCGTAATCTGCTGC<br>TTGCAAACAAAAAAACCACCGCTACCAGCGGTGGTTTGTTTGCCGGATCAAGAGCTACCAACTCTTTTTCCGAAG<br>GTAACTGGCTTCAGCAGAGCGCAGATACCAAATACTGTCCTTCTAGTGTAGCCGTAGTTAGGCCACCACTTCAAG<br>AACTCTGTAGCACCGCCTACATACCTCGCTCTGCTAATCCTGTTACCAGTGGCTGCTGCCAGTGGCGATAAGTCG<br>TGTCTTACCGGGTTGGACTCAAGACGATAGTTACCGGATAAGGCGCAGCGGTCGGGCTGAACGGGGGGTTCGTGC<br>ACACAGCCCAGCTTGGAGCGAACGACCTACACCGAACTGAGATACCTACAGCGTGAGCTATGAGAAAGCGCCACG<br>CTTCCCGAAGGGAGAAAGGCGGACAGGTATCCGGTAAGCGGCAGGGTCGGAACAGGAGAGCGCACGAGGGAGCTT<br>CCAGGGGGAAACGCCTGGTATCTTTATAGTCCTGTCGGGTTTCGCCACCTCTGACTTGAGCGTCGATTTTTGTGA<br>TGCTCGTCAGGGGGGCGGAGCCTATGGAAAAACGCCAGCAACGCGGCCTTTTTACGGTTCCTGGCCTTTTGCTGG<br>CCTTTTGCTCACATACGTAAGTACTCCTGATGCGGTATTTTCTCCTTACGCATCTGTGCGGTATTTCACACCGCA<br>TATATGGTGCACTCTCAGTACAATCTGCTCTGATGCCGCATAGTTAAGCCAGTATACACTCCGCTATCGCTACGT<br>GACTGGGTCATGGCTGCGCCCCGACACCCGCCAACACCCGCTGACGCGCCCTGACGGGCTTGTCTGCTCCCGGCA<br>TCCGCTTACAGACAAGCTGTGACCGTCTCCGGGAGCTGCATGTGTCAGAGGTTTTCACCGTCATCACCGAAACGC<br>GCGAGGCAGCTGCGGTAAAGCTCATCAGCGTGGTCGTGAAGCGATTCACAGATGTCTGCCTGTTCATCCGCGTCC<br>AGCTCGTTGAGTTTCTCCAGAAGCGTTAATGTCTGGCTTCTGATAAAGCGGGCCATGTTAAGGGCGGTTTTTTCC<br>TGTTTGGTCACTGATGCCTCCGTGTAAGGGGGATTTCTGTTCATGGGGGTAATGATACCGATGAAACGAGAGAGG<br>ATGCTCATGCAGGAGCTCGTTTAAAGACAGGAT**GAGACC**
BSmetY	GACGAGGATATCGGCTATCCTAATACGACTCACTATAGGGGAATTGTGAGCGGATAACAATTCCCCTCTAGGAAT<br>AATTTTGTTTAACTTTAAGAAGGAGATATAGACATGAGCAACGAACAGACTTTCCGTCCGGAGACACTTGCAATT<br>CACGCAGGACAGAAACCGGATGCAGAAACCGGTGCACGCGCAGTTCCGATTTACCAGACTAGCAGCTATGTATTC<br>CGTGACAGCGAGCACGCAGCAAACTTATTTGGTTTAAAAGAGGAAGGATTTATTTACACCCGTATTATGAATCCG<br>ACTAATGATGTTTTAGAAAAACGCATTGCAGCACTTGAAGGTGGTATTGGAGCACTTGCATTATCGAGCGGACAA<br>GCAGCAGTTTTTTATAGCATTATTAATATTGCAAGCGCAGGTGATGAAATTGTTAGCAGCAGCTCCATTTACGGT<br>GGAACCTACAATTTATTCGCACATACCTTACGTAAGTTCGGTATTACCGTTAAGTTTGTTGATCCGTCCGATCCG<br>GAAAATTTTGAGCGCGCAATTACTGACAAAACCAAAGCATTATTTGCAGAAACCATTGGTAATCCGAAAAATGAT<br>GTTCTTGACATTGAAGCAGTTGCAGACATTGCACATCGTCATGCAATTCCGCTTATTGTTGACAATACCGTTGCA

	TCGCCATACTTATTACGCCCGATTGAATTTGGTGCAGATATTGTTGTTCACTCGGCAACCAAGTTCATTGGTGGA<br>CACGGTAACAGCATTGGTGGGGTTATTGTTGACAGCGGTAAGTTCGACTGGAAAGGAAGCGGTAAGTTTCCGGAG<br>TTCACTGAGCCAGACCCAAGCTACCACGGATTAGTTTATGTTGACGCAGTTGGTGAAGCAGCATACATTACCAAA<br>GCTCGTATTCAACTTTTACGTGACTTAGGAGCAGCTTTAAGCCCGTTTAACGCATTTTTACTTTTACAGGGATTA<br>GAGACACTTCACTTACGTATGCAAAGACATAGCGAAAACGCACTTGCTGTCGCTAAGTTTTTAGAAGAGGAAGAA<br>GCAGTTGAAAGCGTTAATTACCCAGGACTTCCGAGCCATCCGAGCCATGAATTAGCAAAAAAGTATTTACCGAAC<br>GGACAAGGTGCAATTGTTACCTTTGAAATTAAAGGTGGTGTTGAAGCAGGAAAAAAATTAATTGACAGCGTTAAG<br>TTATTCAGCCATTTAGCAAATATTGGTGATAGCAAAAGCCTTATTATTCACCCAGCAAGCACCACCCATGAGCAA<br>TTAACTCCGGAAGAACAATTATCCGCAGGTGTTACTCCAGGTCTTGTTAGATTATCCGTTGGTACTGAAGCAATT<br>GACGATATTTTAGACGACTTACGTCAGGCAATTCGTCAGAGCCAAACCGTTGGAGTTAAGTAACTAGCATAACCC<br>CTTGGGGCCTCTAAACGGGTCTTGAGGGGTTTTTTGAACTGCTTGGATCC
DRmetX	AGATATGCATGCCGATCCCGCGAAATTAATACGACTCACTATAGGGGAATTGTGAGCGGATAACAATTCCCCTCT<br>AGAAATAATTTTGTTTAACTTTAAGAAGGAGATATACATATGACCGCAGTTCTGGCAGGTCATGCATCGGCACTG<br>CTGCTGACCGAGGAGCCGGATTGCAGCGGACCGCAGACCGTGGTACTGTTTCGTCGAGAACCGCTGCTGCTGGAT<br>TGTGGGCGTGCACTGAGCGATGTTCGTGTTGCATTCCATACCTATGGCACCCCGCGTGCAGATGCAACCCTGGTT<br>CTGCACGCACTGACCGGTGATAGCGCAGTTCACGAATGGTGGCCGGATTTCCTGGGTGCAGGTCGTCCCCTGGAC<br>CCGGCTGATGATTATGTTGTTTGTGCAAATGTGCTGGGTGGATGTGCAGGCACCACCAGCGCAGCGGAGCTGGCA<br>GCAACCTGCTCCGGGCCGGTTCCGCTGAGTCTGCGTGATATGGCACGTGTTGGACGTGCACTGCTGGACTCGCTG<br>GGTGTTAGGCGTGTTCGTGTGATTGGTGCAAGCATGGGTGGAATGCTGGCCTATGCATGGCTGCTGGAATGTCCG<br>GATCTGGTTGAGAAAGCAGTTATCATAGGGGCACCGGCACGTCATAGCCCGTGGGCTATCGGGCTGAATACCGCA<br>GCACGTAGCGCAATCGCACTGGCTCCGGGTGGTGAAGGACTGAAAGTTGCACGTCAGATCGCAATGCTGTCGTAT<br>CGTAGCCCGGAGAGCCTAAGCCGTACCCAGGCAGGACAGCGTGTTCCGGGAGTTCCGGCAGTAACCTCGTATCTG<br>CATTATCAAGGTGAGAAGCTGGCAGCACGTTTTGATGAACAGACCTATTGTGCACTGACCTGGGCAATGGATGCA<br>TTCCAGCCGAGCAGCGCCGATCTGAAGGCAGTTCGTGCACCGGTACTGGTGGTGGGTATTTCCAGCGACCTGCTG<br>TATCCGGCAGCAGAAGTGCGTGCATGTGCAGCAGAACTACCGCACGCAGATTATTGGGAGCTGGGTAGCATCCAC<br>GGTCACGATGCATTCCTAATGGACCCCCAGGATCTACCGGAACGTGTTGGAGCATTCCTGAGGTCGTAACTAGCA<br>TAACCCCTTGGGGCCTCTAAACGGGTCTTGAGGGGTTTTTTGGACGTCCACTGACAGCATCATGGACGA
metX L218A	**CATATG**ACCGCAGTTCTGGCAGGTCATGCATCGGCACTGCTGCTGACCGAGGAGCCGGATTGCAGCGGACCGCAG<br>ACCGTGGTACTGTTTCGTCGAGAACCGCTGCTGCTGGATTGTGGGCGTGCACTGAGCGATGTTCGTGTTGCATTC<br>CATACCTATGGCACCCCGCGTGCAGATGCAACCCTGGTTCTGCACGCACTGACCGGTGATAGCGCAGTTCACGAA<br>TGGTGGCCGGATTTCCTGGGTGCAGGTCGTCCCCTGGACCCGGCTGATGATTATGTTGTTTGTGCAAATGTGCTG<br>GGTGGATGTGCAGGCACCACCAGCGCAGCGGAGCTGGCAGCAACCTGCTCCGGGCCGGTTCCGCTGAGTCTGCGT<br>GATATGGCACGTGTTGGACGTGCACTGCTGGACTCGCTGGGTGTTAGGCGTGTTCGTGTGATTGGTGCAAGCATG<br>GGTGGAATGCTGGCCTATGCATGGCTGCTGGAATGTCCGGATCTGGTTGAGAAAGCAGTTATCATAGGGGCACCG<br>GCACGTCATAGCCCGTGGGCTATCGGGCTGAATACCGCAGCACGTAGCGCAATCGCACTGGCTCCGGGTGGTGAA<br>GGACTGAAAGTTGCACGTCAGATCGCAATGCTGTCGTATCGTAGCCCGGAGAGCGCGAGCCGTACCCAGGCAGGA<br>CAGCGTGTTCCGGGAGTTCCGGCAGTAACCTCGTATCTGCATTATCAAGGTGAGAAGCTGGCAGCACGTTTTGAT<br>GAACAGACCTATTGTGCACTGACCTGGGCAATGGATGCATTCCAGCCGAGCAGCGCCGATCTGAAGGCAGTTCGT<br>GCACCGGTACTGGTGGTGGGTATTTCCAGCGACCTGCTGTATCCGGCAGCAGAAGTGCGTGCATGTGCAGCAGAA<br>CTACCGCACGCAGATTATTGGGAGCTGGGTAGCATCCACGGTCACGATGCATTCCTAATGGACCCCCAGGATCTA<br>CCGGAACGTGTTGGAGCATTCCTGAGGTCGTAACTAGCATAACCCCTTGGGGCCTCTAAACGGGTCTTGAGGGGT<br>TTTTTG**GACGTC**

**Name**	**Amino acid sequence (N→C terminus)**
MetX	MTAVLAGHASALLLTEEPDCSGPQTVVLFRREPLLLDCGRALSDVRVAFHTYGTPRADATLVLHALTGDSAVHEW<br>WPDFLGAGRPLDPADDYVVCANVLGGCAGTTSAAELAATCSGPVPLSLRDMARVGRALLDSLGVRRVRVIGASMG<br>GMLAYAWLLECPDLVEKAVIIGAPARHSPWAIGLNTAARSAIALAPGGEGLKVARQIAMLSYRSPESLSRTQAGQ<br>RVPGVPAVTSYLHYQGEKLAARFDEQTYCALTWAMDAFQPSSADLKAVRAPVLVVGISSDLLYPAAEVRACAAEL<br>PHADYWELGSIHGHDAFLMDPQDLPERVGAFLRS*
MetY	MSNEQTFRPETLAIHAGQKPDAETGARAVPIYQTSSYVFRDSEHAANLFGLKEEGFIYTRIMNPTNDVLEKRIAA<br>LEGGIGALALSSGQAAVFYSIINIASAGDEIVSSSSIYGGTYNLFAHTLRKFGITVKFVDPSDPENFERAITDKT<br>KALFAETIGNPKNDVLDIEAVADIAHRHAIPLIVDNTVASPYLLRPIEFGADIVVHSATKFIGGHGNSIGGVIVD<br>SGKFDWKGSGKFPEFTEPDPSYHGLVYVDAVGEAAYITKARIQLLRDLGAALSPFNAFLLLQGLETLHLRMQRHS<br>ENALAVAKFLEEEEAVESVNYPGLPSHPSHELAKKYLPNGQGAIVTFEIKGGVEAGKKLIDSVKLFSHLANIGDS<br>KSLIIHPASTTHEQLTPEEQLSAGVTPGLVRLSVGTEAIDDILDDLRQAIRQSQTVGVK*
MetX*	MTAVLAGHASALLLTEEPDCSGPQTVVLFRREPLLLDCGRALSDVRVAFHTYGTPRADATLVLHALTGDSAVHEW<br>WPDFLGAGRPLDPADDYVVCANVLGGCAGTTSAAELAATCSGPVPLSLRDMARVGRALLDSLGVRRVRVIGASMG<br>GMLAYAWLLECPDLVEKAVIIGAPARHSPWAIGLNTAARSAIALAPGGEGLKVARQIAMLSYRSPESASRTQAGQ<br>RVPGVPAVTSYLHYQGEKLAARFDEQTYCALTWAMDAFQPSSADLKAVRAPVLVVGISSDLLYPAAEVRACAAEL<br>PHADYWELGSIHGHDAFLMDPQDLPERVGAFLRS*

L218A mutation in MetX is highlighted in red.

Table S3. Composition of the trace element solution. All ingredients were dissolved in 5 M HCl at the indicated concentration.

Component	Concentration [g/L]
$FeSO_4 \cdot 7\ H_2O$	40
$MnSO_4 \cdot H_2O$	10
$AlCl_3 \cdot 6\ H_2O$	10
$CoCl_2 \cdot 6\ H_2O$	7.3
$ZnSO_4 \cdot 7\ H_2O$	2
$Na_2MoO_4 \cdot 2\ H_2O$	2
$CuCl_2 \cdot 2\ H_2O$	1
H_3BO_3	0.5

Table S4. Composition of the batch medium for 10 g/L CDM. All ingredients were filled up to a total weight of 400 g with H_2O. Trace element solution composition is detailed in Table S3.

Component	Amount	
KH_2PO_4	2.09	g
85% (w/w) H_3PO_4	0.71	g
Yeast Extract	0.60	g
Na-citrate dihydrate	0.16	g
$MgCl_2 \cdot 6\ H_2O$	0.18	g
$CaCl_2 \cdot 2\ H_2O$	0.08	g
Trace element solution	0.20	mL
$(NH_4)_2SO_4$	2.95	g
D-glucose $\cdot$ H_2O	12.12	g

Table S5. Composition of the fed-batch medium. All ingredients were filled up to a total weight of 835 g with H2O.

Component	Amount	
KH_2PO_4	4.02	g
85% (w/w) H_3PO_4	1.36	g
Na-citrate dihydrate	2.51	g
$MgCl_2 \cdot 6\ H_2O$	2.81	g
$CaCl_2 \cdot 2\ H_2O$	1.23	g
Trace element solution	3.05	mL
$(NH_4)_2SO_4$	2.95	g
D-glucose · H_2O	184.85	g

Table S6. Strains and plasmids used in this study.

***E. coli* strain**	**Plasmid**	**Expression of *metXY***	**Growth & protein production**
BL21(DE3){pXYI}	pXYI	bicistronic	not decoupled
BL21(DE3){pXYII}	pXYII	monocistronic	not decoupled
BL21(DE3){pXYIII}	pXYIII	monocistronic	not decoupled
e^Xpress V1{pXYI}	pXYI	bicistronic	decoupled
e^Xpress V1{pXYII}	pXYII	monocistronic	decoupled
e^Xpress V1{pXYIII}	pXYIII	monocistronic	decoupled
e^Xpress V2{pXYI}	pXYI	bicistronic	decoupled
e^Xpress V2{pXYII}	pXYII	monocistronic	decoupled
e^Xpress V2{pXYIII}	pXYIII	monocistronic	decoupled
e^Xpress V1{pX*YIII}	pX*YIII	monocistronic	decoupled
